# Collaborative and selfish mitigation strategies to tackle energy scarcity: The case of the European gas crisis

**DOI:** 10.1016/j.isci.2023.106750

**Published:** 2023-04-27

**Authors:** Jacob Mannhardt, Paolo Gabrielli, Giovanni Sansavini

**Affiliations:** 1 Institute of Energy and Process Engineering, ETH Zurich, 8092 Zurich, Switzerland; 2 Department of Global Ecology, Carnegie Institution for Science, Stanford, CA, USA

**Keywords:** Energy resources, Energy policy, Energy systems

## Abstract

Following the disruption of Russian natural gas flows to Europe, we investigate the impact of collaborative and selfish behavior of European countries to tackle energy scarcity and supply electricity, heat, and industrial gas to end users. We study how the operation of the European energy system will need to adapt to the disruption and identify optimal strategies to overcome the unavailability of Russian gas. Those strategies include diversifying gas imports, shifting energy generation to non-gas-based technologies, and reducing energy demands. Findings suggest that: (1) selfish behavior of Central European countries exacerbates the energy scarcity for many Southeastern European countries; (2) proactive collaborative energy savings, together with a mild winter, can fully relieve the stress of the gas shortage; (3) diversification of gas imports leads to bottlenecks in the gas network, especially in Southeastern Europe; and (4) electricity generation is mostly shifted to coal-based power plants, causing higher carbon emissions.

## Highlights

• Assessing the energy crisis in Europe after the disruption of Russian natural gas
• Selfish behavior of European countries exacerbates the crisis for Eastern Europe
• Then, significant involuntary electricity and heat demand reductions are necessary
• Diversification of gas imports leads to bottlenecks in the European gas network

## Graphical abstract

**Figure:**
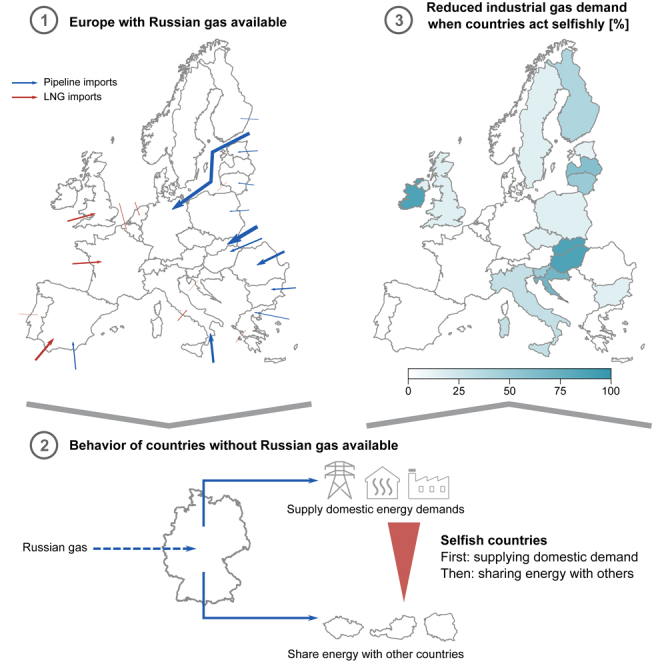

## Introduction

Europe is in the tight grip of the worst energy crisis in decades.^1^ Following Russia’s invasion of Ukraine and the resulting sanctions by European countries, Russia drastically reduced gas exports to Europe. In 2021, the European Union (EU) imported 155 billion cubic meters (bcm, around 1500 TWh) of natural gas from Russia, accounting for almost 40% of its total gas consumption.^2^ In November 2022, imports were reduced by over 80%, with only two pipelines being in partial operation.^3^ The perspective of a gas shortage during winter led to soaring energy prices, and most European citizens, policymakers, and companies worry about how to get safely through this and possibly future energy crises.

Natural gas is primarily consumed to provide energy services, i.e., electricity, residential and commercial heat, and industrial services.^4^ Hence, answering the question of how to overcome the energy crisis translates to understanding under which conditions a supply shortage of gas leads to the least harmful reductions in final energy services.

Assessing the current situation is especially challenging because of the multitude of possible levers to compensate the gas shortage. These include increasing non-Russian gas imports, shifting to non-gas-based electricity and heat generation, and reducing gas consumption through voluntary and involuntary demand reduction mechanisms. Various aspects of the current gas shortage in Europe have been investigated in the scientific literature, namely, the economic assessment of Russia’s market power,^5^ the technical feasibility of operating the European gas network under a gas shortage,^6^ and possible strategies to compensate the gas shortage.^7^^,^^8^ All presented European models assume that all countries will act perfectly collaborative to follow the optimal strategies.^5^^,^^6^^,^^7^

However, in times of crisis, countries tend to put their self-interest before the common interest of the international community.^9^ The European gas supply network is highly interconnected and transboundary with few import points for the entire continent. Thus, most countries depend on European collaboration for gas supply.^10^ Fatih Birol, executive director of the International Energy Agency (IEA), called the upcoming winter “a historic test of European solidarity, one it cannot afford to fail”.^11^ During the energy crisis, some European countries have declared to or have been accused of reducing their energy exports to ensure domestic energy supply.^12^^,^^13^^,^^14^^,^^15^^,^^16^ During the energy crisis, selfishly acting countries will first supply their domestic energy demands before they share gas and electricity with neighboring countries. The exchange of energy between countries has been investigated in the past by enforcing the autarky of regions.^17^^,^^18^ However, the current energy crisis is unprecedented in that for the first time exchanging energy among countries might directly impact domestic security of supply. Thus, the existing approaches are not equipped to investigate selfish behavior under which exports are restricted to ensure the domestic security of energy supply. Enforcing energy autarky is not the same rationale as withholding energy from others to supply domestic demand. To the best of our knowledge, selfish behavior among countries to tackle energy scarcity has not yet been investigated.

European countries have shown and continue to make concessions to mitigate the impact of the gas shortage on the electricity, heating, and industry sectors.^19^ The member states of the EU agreed on a voluntary gas demand reduction target of 15% compared to their average gas consumption of the preceding five years.^20^ The European Commission proposes various measures for all three sectors to voluntarily reduce the gas demand, including fuel switching in the industry and thermostat adjustment in residential and commercial heating.^10^ If voluntary reduction measures prove insufficient, the demand must then be further reduced involuntarily through governmental intervention.^20^ All studies investigating the current energy crisis assume exogenous reductions of final energy demands, thus, making *a-priori* assumptions about what demand reductions will materialize.^5^^,^^6^^,^^7^^,^^8^ Hence, they cannot provide optimal strategies to balance voluntary and involuntary demand reductions.

The objective of all presented studies of the energy crisis is to close the gas balance, i.e., match supply and demand of gas under a Russian gas disruption. However, most works only investigate the gas sector in isolation, neglecting the electricity and heating sector.^5^^,^^6^^,^^8^ Excluding other sectors neglects that the options of either shifting to non-gas-based generation or reducing the final energy demand have divergent implications on the energy system, though they potentially lead to comparable reductions in natural gas consumption. Some authors take the degrees of freedom in the electricity sector into account and investigate the possibility of shifting from gas-based electricity generation to hard coal power plants by linking an hourly resolved electricity sector model with a monthly resolved gas sector model.^7^

Overall, we fill these gaps by investigating the risk of energy scarcity in Europe through the lens of selfish versus collaborative behavior of European countries. We do so by considering the entire gas supply chains, from increased imports of natural gas to shifts in the electricity and heat generation mix and necessary reductions of final energy demands. More specifically, we extend the existing studies by addressing the following questions.

1. How much do the European countries need to voluntarily and involuntarily reduce the electricity, residential and commercial heat, and industrial gas demand under a Russian gas disruption if they act collaboratively or selfishly?
2. How are the gas flows between countries redistributed because of the diversification of gas imports? Is the European gas flow network capable of accommodating the redistributed flows?
3. What are strategies to substitute the missing Russian gas imports in Europe? To what extent can the coupling of the gas, electricity, and heating sectors support the reduction of gas consumption by shifting energy generation to non-gas-based technologies?
4. To what extent do proactive energy savings and a mild or severe winter impact Europe’s ability to mitigate the gas shortage?
5. How can the energy system prepare for the following winter while supplying the energy demands as much as possible during the year without Russian gas to fill the gas storages?

We tackle these questions through a European energy system model that optimizes the operation of the gas, electricity, and heating sectors in combination for 28 European countries to assess the impact of a complete disruption of the Russian gas supply. The objective of the optimizer is to minimize the total social welfare cost of the energy system, which is composed of the cost to operate the technologies to supply final energy demands and of the cost for endogenous demand reductions if the demands cannot be fully supplied. The exchange of energy is modeled as an electricity and gas transfer model between the countries. This sector-coupled model is described in STAR Methods and allows quantifying the optimal mix of electricity and heat generation under a gas shortage, and the optimal, necessary final energy demand reductions. To model the operation of the energy system in detail while ensuring computational tractability, we aggregate the hourly resolved input data to 1,400 representative time steps (see Experimental procedure S4).

We assess the different adaption strategies if the European countries act in a collaborative spirit or selfishly. In detail, our definition of selfish behavior is that countries will first attempt to supply their entire final energy demands before they share any excess gas and electricity with neighboring countries. To the best of our knowledge, the presented approach is the first centrally planned energy system optimization model which allows the investigation of selfish behavior of individual countries to tackle energy scarcity.

Unlike other studies, we treat the demand reduction as an endogenous choice of the optimizer, thus we are able to indicate where and when to reduce the electricity, heat, and industrial gas demand to maximize social welfare. To assess the real-world consequences of reducing final energy demands, and thus the impact of the energy crisis, we differentiate between voluntary and involuntary demand reductions. To this end, we develop a modeling approach where the energy demand of each sector can be reduced on two levels: The first, voluntary reduction level leads to lower costs than the second, involuntary level. However, voluntary demand reductions are limited to a share of the demand. After reducing the entire voluntary share, if still necessary, the demand must be reduced on the second, involuntary level at higher costs. STAR Methods detail the share, costs, and the ensuing priority order of the voluntary and involuntary demand reductions for each energy sector. Although the numerical value of costs is somewhat arbitrary, it does not impact the results of the optimization as long as the priority order of demand reductions is maintained, because demand reductions are always the ultima ratio and thus do not compete with other mitigation strategies. The sensitivity of the parameters is qualitatively assessed in Table S11.

Finally, this study focuses on short-term techno-economic mitigation strategies and does not emphasize the macroeconomic feedback of the energy crisis.

## Results

This study provides a cost-optimal pathway through the energy crisis by investigating all three mitigation strategies along the entire gas supply chain.

(i) Additional gas imports from other sources than Russia;
(ii) shift of electricity and heat generation to non-gas-fired technologies;
(iii) reduction of final energy demands.

We first analyze the demand reductions in the electricity, heating, and industrial sector as these reductions are the direct impact of the gas shortage and thus quantify how well the energy crisis can be overcome if all countries collaborate. Subsequently, we contrast collaborative with selfish behavior where each country first attempts to fully supply its domestic energy demands. In all presented results, the gas storage levels at the end of the year must match the initial levels for each country to ensure that the energy system is prepared for the winter of 2023.

### Collaborative and selfish demand reductions

**Figure 1:**
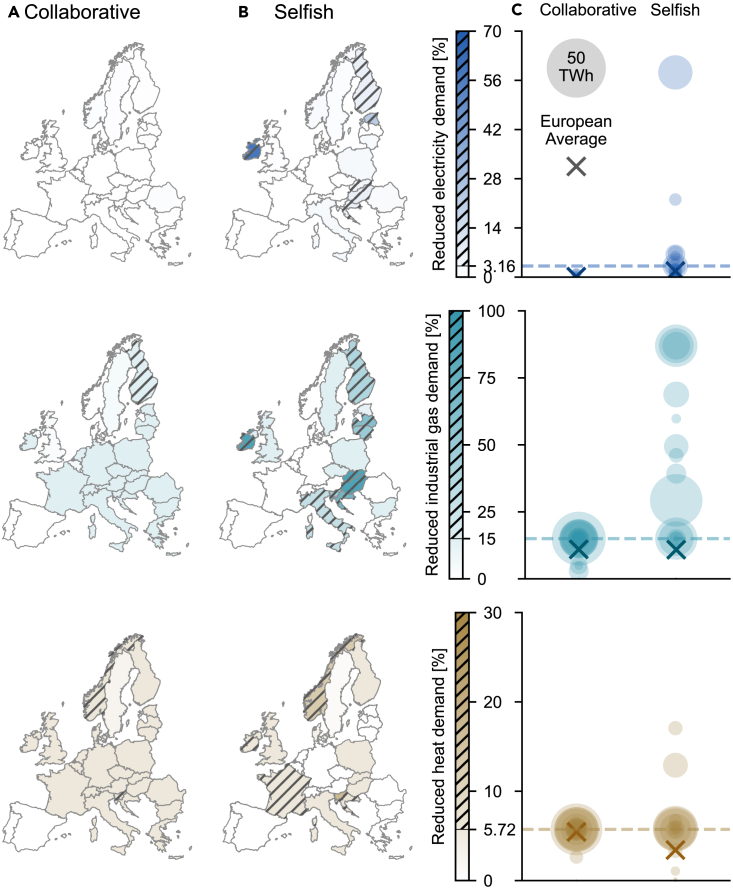
Annual reduction of final energy demands under collaborative and selfish action (A–C) Annual reduction of electricity, industrial gas, and heat demand to adapt to a Russian gas shortage under collaborative (A) and selfish behavior (B) from November 1, 2022, until October 31, 2023. Countries with involuntarily reduced demand are hatched. Spread of annual demand reductions of all countries (C). The size of the circles indicates the absolute demand reduction of each country in each sector; the center is located at the corresponding relative demand reduction. The limit between voluntary and involuntary demand reduction is indicated by dashed lines. The dark crosses indicate the average demand reductions across all European countries.

Figure 1 shows the necessary annual demand reductions of each country in the European electricity, industrial gas, and heating sector, and the spread of demand reductions across the countries. In the collaborative scenario (Figure 1A), all countries support each other by reducing their own demand and subsequently sharing gas and electricity, resulting in nearly uniform demand reductions across the countries (left side of Figure 1C). In particular, most countries voluntarily reduce a share of their industrial gas and heat demand at comparatively little harm. Only a few countries show annual involuntary demand reductions in any energy sector. These are Finland in the industrial gas sector and Norway and Slovenia in the heating sector. Figure 1 shows that the three countries with involuntary demand reductions overshoot the voluntary limit by only a few percentage points, which are too small to be detectable in Figure 1C.

The electricity demand is the last to be reduced voluntarily and multiple technologies exist to replace gas-based electricity generation (as detailed in Figure 4 and Table S1), therefore, most countries can fully supply their electricity demand and the average European demand reduction is close to zero (Figure 1C). Romania is the only country that cannot fully supply its electricity demand. Most countries reduce exactly the voluntary quantity of industrial gas. Only the Iberian Peninsula, the UK, Norway, and Sweden can supply their industrial gas demand to a higher degree because of abundant access to gas imports. In the heating sector, the reduced demand levels are almost uniformly at the voluntary limit. Again, the Iberian Peninsula can supply its entire heat demand. The spread between the remaining countries is negligible with only Sweden well below the voluntary threshold. The average European demand reduction for heat and industrial gas is close to the voluntary limit (Figure 1C).

In times of crisis, however, countries tend to value their domestic energy supply above the common interest of the international community.^12^^,^^13^^,^^14^ We model the selfish behavior of individual countries via a binary decision-making process: The countries decide between either exporting gas and electricity or reducing final energy demands voluntarily. If they export energy, all domestic demand reductions are involuntary. In this way, a country has no incentive to share its energy with other countries when sharing energy implies reducing its domestic demands. Thus, if the countries act selfishly, they prioritize fully supplying their domestic demand and do not support other countries anymore by voluntarily reducing their own demand to share gas and electricity. The STAR Methods detail the binary decision-making.

The right side of Figure 1C shows that the amounts of reduced energy demands spread much more when countries adopt a selfish behavior than when they act collaboratively. Hence, selfish behavior leads to an imbalance between countries that have the power to fully supply their energy demands and those that rely on energy imports from other countries. Figure 1B shows that Central European countries such as Germany, Belgium, and the Netherlands can supply all their final energy demands by acting selfishly, which exacerbates the energy crisis for many countries in Eastern and Southeastern Europe; for example, Croatia, Hungary, and Slovenia. Countries in need of energy imports are forced to involuntarily reduce their energy demands by up to 58% (electricity), 87% (industrial gas), and 17% (heat). Hence, by trying to supply their domestic energy demands as much as possible, countries cause involuntary demand reductions in other countries. This trend is exacerbated when investigating the demand reductions for each hour of the year (Figure S1). In the selfish scenario, high heat and industrial gas demands are fully supplied more often, which leads to strong involuntary reductions at lower demands.

Gas is not only shared among countries but also between the three sectors, which leads to a trade-off in gas distribution: When acting selfishly, many Central European countries consume more gas to generate heat because of the lack of alternative technologies, so the reduction of heat demand in Europe is less pronounced. The reduction of industrial gas demand slightly decreases as well. These quantities of gas are then missing in the electricity sectors of other countries. Hence, the average reduction of electricity demand in Europe increases. In the collaborative scenario, the order in which the final energy demands are reduced (STAR Methods) applies to all countries in combination. Therefore, if the countries collaborate, they first try to reduce their heat demand all together until the voluntary limit is reached in all countries, followed by the industrial gas demand, and lastly the electricity demand. This collaborative effort to remain within the voluntary limit leads to the almost uniform demand reductions across countries observed in Figure 1A. The small violations of the voluntary limit by Norway, Finland, and Slovenia in the collaborative scenario stem from insufficient transport capacities during a few hours of the year. In contrast, when acting selfishly, the order of demand reductions applies to each country individually. For example, some countries must reduce their electricity demand involuntarily before the heat demand is voluntarily reduced in other countries.

Voluntary demand reductions are considered less harmful than involuntary reductions. Increasing involuntary demand reductions in some countries and sectors to decrease voluntary demand reductions in other countries and sectors leads to a more harmful impact of the gas shortage. In reality, countries will most likely adopt an intermediate approach between collaborative and selfish behavior, therefore, our results demonstrate the extremes of the heterogeneous decision-making spectrum.

### Reversal of gas flow corridors in Europe

The European gas network is strongly interconnected and transboundary and evolved to serve the import of natural gas from Russia. We investigate the shift in gas flows caused by the unavailability of Russian gas and the extent to which the European gas flow network can accommodate the redistribution of gas from other sources.

**Figure 2:**
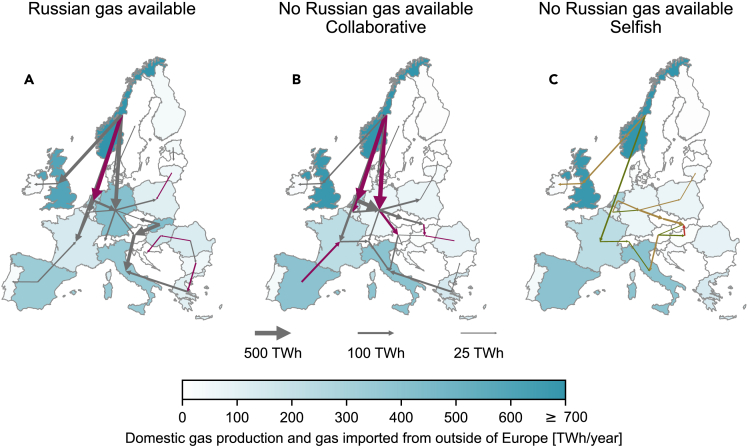
Annual gas flows in Europe with Russian gas available and unavailable (A–C) Annual gas flows in Europe with Russian gas available (A) and unavailable (B and C); collaborative (B) and selfish behavior (C). The arrows in (A) and (B) indicate net annual flows; purple arrows highlight fully utilized cross-border capacities (shown for utilization of transport capacity ≥ 95%). The arrows in (C) show the differences in flows between the selfish (C) and the collaborative (B) scenarios. Green: gas flows increase from collaborative to selfish scenario, and maintain the same direction. Brown: gas flows decrease from collaborative to selfish scenario, but maintain the same direction. Red: gas flows decrease from collaborative to selfish scenario, and change direction. Only flows larger than 2.5% of the maximum flow are shown. The annual domestic gas production and the gas imports from outside of Europe are shown through the colorbar. Norway produces around 1200 TWh of gas annually.

Figure 2 shows the domestic gas production and the imported gas from outside Europe, and the flows between countries if (1) Russian gas is still available, (2) no Russian gas is available and the countries act collaboratively, and (3) no Russian gas is available and the countries act selfishly.

With Russian gas available, the sources of natural gas are distributed across Europe with a substantial amount entering the energy system from the Northeast (Figure 2A). The largest source is Norway with domestic production of approximately 1200 TWh annually, followed by the UK with around 700 TWh annually. Figure S2 shows a detailed breakdown of the gas sources for each country.

The main importers of Russian gas are Germany and Slovakia. There are therefore three main gas flow corridors (1)–(3) when Russian gas is available (Figure 2A): (1) Norway supplies the western side of Europe with large flows to Germany, the UK, and the Netherlands, which are then transported further to Belgium and France. In addition to the Norwegian supply, Germany is mainly supplied by Russia. After consuming 85% of the supplied gas, Germany exports the remainder, mainly to Switzerland, Austria, and Poland. (2) The second corridor consists of gas entering Slovakia. These gas imports are mainly transported to Italy, one of the main European gas consumers, via Austria. A smaller share of natural gas flows from Slovakia to the Czech Republic. (3) Russian gas imports into Romania supply the Balkan region and Greece, from where additional gas is further transported to Italy. As the gas infrastructure is designed for this flow pattern, only a few cross-border connections are utilized at maximum capacity (purple arrows in Figure 2A).

Without Russian gas, Eastern European countries and Germany can neither import gas via pipelines from outside Europe nor count on sufficient domestic production (Figure S2). Those countries that historically have been the main entry points of Russian gas are now in need of gas supplies from other countries. This geographic shift in imports leads to a redistribution of gas flows to Germany and Eastern Europe. The general flow direction with Russian gas available is from Northeast to Southwest (Figure 2A). Without Russian gas, the general flow direction is from Northwest to Southeast if the countries act collaboratively (Figure 2B); in this case, Germany relies on Norway, the Netherlands, and Belgium to replace Russian gas and meet its energy demands. This results in fully utilized cross-border capacities from Norway and the Netherlands to Germany (purple arrows in Figure 2B).

The flows to the Southwest are reduced so that the corridors to France and to Italy carry less gas. The flow from Austria to Italy is almost entirely reduced, which is compensated for by additional flows from Switzerland to Italy. To this end, much more gas is transported eastward from Spain via France to Switzerland. The new gas corridor is formed from Germany to the East and Southeast, which leads to several cross-border capacities in Central Europe being fully utilized, i.e., from Germany to Austria, and from Slovakia and Romania to Hungary. The cross-border capacity between Spain and France becomes fully utilized because Spain is the country with the most abundant access to natural gas through pipelines from Northern Africa and liquefied natural gas (LNG) imports. Because this cross-border capacity is the only gas flow connection from Spain to Central Europe, Spain cannot increase gas exports to further support other countries.

The flow pattern in the selfish scenario is similar to the one in the collaborative scenario, although the flows decrease (the arrows in Figure 2C show the difference in annual flows between the selfish and the collaborative scenario). In particular, the eastbound flow from the Netherlands through Germany, the Czech Republic, and Slovakia to Hungary and the flow from Austria to Italy are significantly reduced. Therefore, the reduced gas exports in the selfish scenario exacerbate the situation for countries in Southeastern Europe (Figure 1).

Overall, the disruption of Russian gas drastically changes the flow pattern in the European gas flow network. With Russian gas available, the gas is mainly imported in the Northeast and transported to the Southwest. When Russian gas becomes unavailable, the main flow direction leads from Northwest to Southeast. As the gas network was not designed for that flow direction, essential cross-border connections are operated at maximum capacity. Increased final energy demands, for example, in a severe winter, then lead to additional demand reductions in Eastern and Southeastern Europe.

### Strategies to mitigate gas shortage

Three short-term compensation strategies can help mitigate the gas shortage.

(i) Additional supply of gas from sources other than Russia. This includes additional pipeline and LNG imports;
(ii) shift to non-gas-based electricity and heat generation, including the reduction of electrified heat;
(iii) reduction of final energy demands.

**Figure 3:**
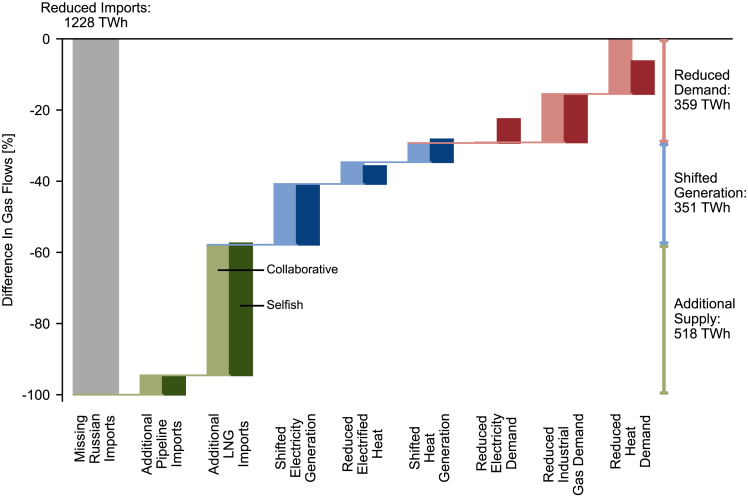
Compensation of missing Russian gas imports under collaborative and selfish action Compensation of missing Russian gas imports in collaborative (light) and selfish (dark) scenario. Transport and storage losses in the gas flow network are allocated to the additional LNG import bar. Shifted electricity generation accounts for the difference in electricity transport and storage losses. All values are in quantities of displaced gas.

Figure 3 shows the contribution of the compensation strategies to mitigate the gas shortage in Europe for the collaborative and the selfish scenario. The missing Russian gas imports add up to around 1250 TWh yearly, which accounts for more than 35% of all imports of natural gas and LNG, and domestic gas production in the scenario with Russian gas available. The total Russian gas imports are lower than the measured value in 2021 (around 1500 TWh^2^), because the scenario with Russian gas available already includes the additional renewable capacities available in 2022, thus lowering the consumption of natural gas.

**Figure 4:**
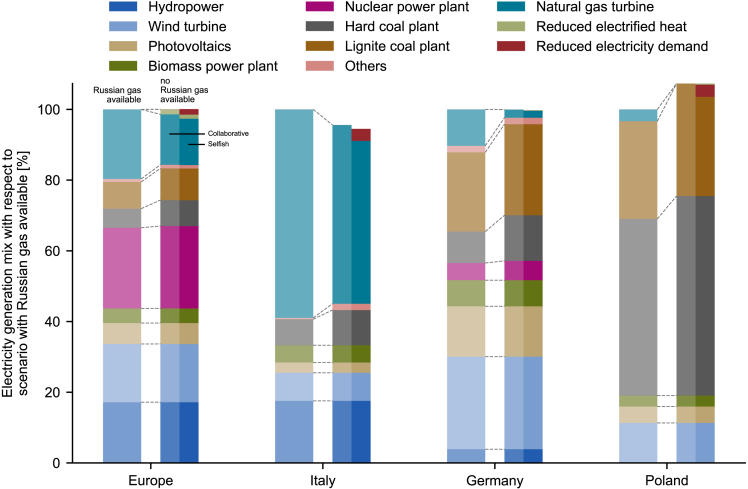
Variation of electricity generation mix from the scenario with and without Russian gas available Variation in electricity generation mix from the scenario with Russian gas available (left bar) to the scenarios without Russian gas (right bar), for collaborative scenario (left side of right bar) and selfish scenario (right side of right bar). The energy mix is scaled to the electricity generation with Russian gas available. The energy generation mix is shown for the whole of Europe, Italy, Germany, and Poland. Hydropower: run-of-river and reservoir hydropower; wind turbine: onshore and offshore wind turbines; others: oil and waste power plants.

In both the collaborative and selfish scenarios, the missing Russian gas imports are replaced by increasing non-Russian gas imports (about 42% of the total missing Russian gas), by increasing the share of non-gas-based electricity and heat generation (about 28%), and by reducing final energy demands (the final 30%). The collaborative and selfish scenarios are almost identical in the absolute amount of replaced gas through additional supply and shifted generation. However, in the selfish scenario, more electricity demand is reduced in Europe whereas the reductions in heat demand decrease (Figure 1).

Non-Russian imports account for around 520 TWh, which is within the range reported by other authors.^19^^,^^24^ Pipeline imports from Northern Africa and Turkey are expected to rise only slightly,^25^ hence providing little contribution (about 5%) to substitute the missing Russian imports; the greatest contribution is given by increased LNG imports (about 37%), with global export capacities being expected to allow around 537 TWh of additional LNG imports to Europe (Experimental procedure S7^25^). In particular, the US is expected to significantly increase its export capacities.^26^ Our results suggest that the existing capacities of LNG terminals and the gas flow network are sufficient to accept most of these additional quantities and distribute the gas to the countries. This excludes Spain, which has the largest LNG import capacities in Europe but cannot effectively deliver the additional LNG to other European countries because of the limited pipeline capacity between Spain and France (Figure 2). With the new LNG infrastructure commissioned in Germany, Poland, Finland, and Estonia in 2022 and January 2023, the LNG import capacities are distributed along the European shore. The spatially diversified import of LNG facilitates the transport of gas across the continent.

The IEA^19^ reports that the gas demand in the EU decreased by around 490 TWh in 2022, which is around 20% less than the reduced demand and shifted generation accounts for in this study. However, it is noted that Russian gas was available over the course of the year and the beginning winter of 2022 was unusually warm. Behavioral action by consumers contributed to reductions in gas consumption of up to 100 TWh in 2022.^19^ This evidence is in line with the overall voluntary and involuntary heat demand reduction in this study. The results suggest that this behavioral action contributes to decreasing EU gas consumption by around 85 TWh in the selfish scenario and 160 TWh in the collaborative scenario.

Figure 4 shows the shift in the electricity generation mix when Russian gas becomes unavailable for the whole of Europe and for relevant countries, namely Italy, Germany, and Poland. The largest share of shifted generation is electricity generated from coal instead of natural gas. There is ample coal power capacity in Europe that is currently idle and can be utilized to compensate for the missing electricity previously generated from gas. However, a large share of that capacity has not generated any electricity in the past year, and thus cannot be brought back to operation in a reasonable time (Experimental procedure S11). Thus, the usable capacity of thermal power plants may be more limited than assumed in other works.^7^

Without Russian gas available, the electricity generation from natural gas in Europe is reduced by around 29% in the collaborative scenario and around 39% in the selfish scenario. Gas-based electricity generation is mainly substituted by increasing the utilization of hard coal (+37%) and lignite power plants (+19%). Electricity generated from nuclear power plants can be increased by around 2%, because very little available nuclear capacity is currently unused. Renewable technologies are always the first to dispatch because of their low marginal cost of production. Hence, they already exploit their entire available capacity and cannot further contribute to compensate for the gas shortage. Substantially higher electricity generation from renewable technologies can only be achieved through additional investments. In the case of selfish behavior, it also becomes necessary to reduce substantial amounts of electricity demand (Figure 1).

The existing electricity generation capacities determine the amount of electricity that can be shifted away from natural gas in each country. With Russian gas available, Italy generates more than half of its electricity from natural gas and does not have large additional idle capacities of other technologies that are available to replace it. Thus, Italy can only reduce its gas-based electricity generation by 16% in the collaborative scenario by exploiting the available idle hard coal capacity, and net imports must be increased (lower absolute height of the bar in Figure 4). In the selfish scenario, less gas is delivered to Italy (Figure 2) which in turn reduces gas-based electricity generation by 23% and, furthermore, must reduce the electricity demand.

Germany and Poland both have significant capacities of coal power plants available. They are able to generate more electricity from non-gas-based technologies with respect to Italy and other European countries. Poland in particular increases the electricity generation from coal to reduce the net electricity import when no Russian gas is available. Thanks to the available coal power capacity, Germany substitutes around 77% of its gas consumption in the electricity sector, with the total amount of generated electricity remaining almost constant.

The potential of shifting residential and commercial heat generation to non-gas-based technologies is strongly limited, and can only substitute the missing imports of Russian gas by around 6% (Experimental procedure S14).

Figure 3 shows that mitigating the gas shortage requires reducing the consumption of electrified heat. Although heat pumps provide around the same amount of heat with and without Russian gas available, the use of electric boilers is strongly reduced. In a tense electricity sector under a gas shortage, all electricity is valuable. Thus, it is more efficient to directly convert gas into heat in gas boilers than to convert gas into electricity through gas turbines first and then use the electricity to produce heat in electric boilers; this does not apply to heat pumps, which feature a much higher conversion efficiency than gas boilers, even considering the efficiency of natural gas turbines to generate electricity. The IEA recommends increasing the electrification of heat, mostly through heat pumps also other electric heating, to reduce the gas consumption in households.^19^ Our results show that heat pumps are used at maximum capacity; however, other types of electric heating are strongly avoided because of their higher strain on the electricity sector.

In summary, the shortage of Russian gas can be fully compensated only through the combination of increased non-Russian imports, shifted electricity and heat generation, and reduced final energy demands. Idle capacities of thermal power plants, especially lignite and hard coal, allow shifting the electricity generation toward non-gas-based technologies to meet final energy demands. However, the shift to coal-based generation increases the electricity sector’s annual carbon emissions by around 24.4 Mt (+5%, IEA estimates 35 Mt higher carbon emissions in the EU power sector in 2022^19^), which diminishes the capability of the electricity sector to comply with climate targets.^27^ Continued and ambitious investments in renewable energy generation, which are arguably catalyzed by the current energy crisis,^28^ will be needed in the future to offset this increase in carbon emissions and mitigate the climate impact of the energy sector.

### Demand reductions in different winter scenarios

Europe’s ability to get through the energy crisis without significant reductions in energy demands strongly depends on the severity of winter. Thus, we investigate three winter scenarios “mild winter and high energy savings”, “winter as usual” and “severe winter” as extreme scenarios on the spectrum of potential changes in final energy demands. In the case of high energy savings and a mild winter, we assume that the heat and electricity demands are 20% and 10% lower, respectively, with respect to the usual winter scenario for all countries at all times.^29^ In contrast, in the severe winter scenario, the heat demand is 20% higher with respect to the usual winter scenario, whereas the electricity demand remains constant. The industrial gas demand does not change across the considered scenarios.

**Figure 5:**
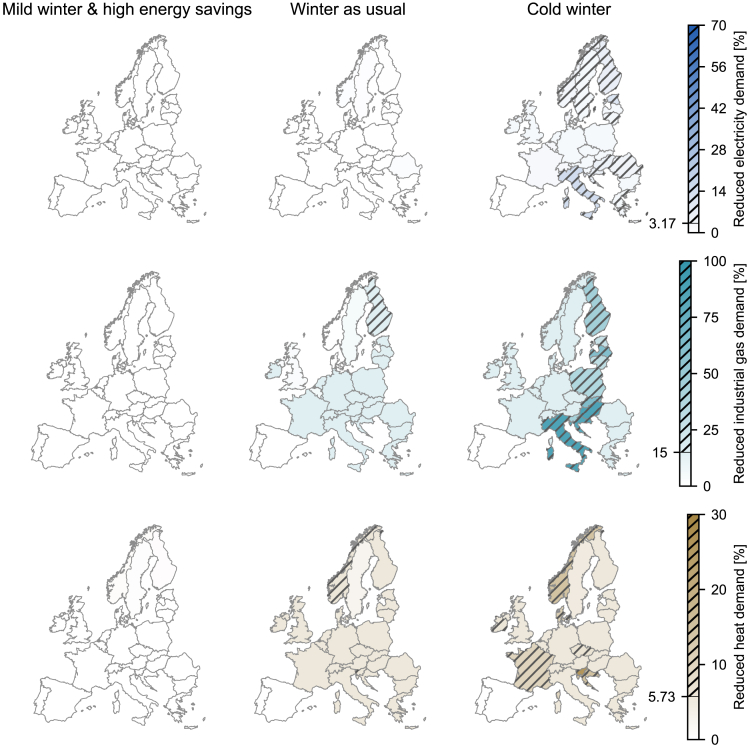
Europe without Russian gas in a mild, reference, and severe winter scenario Europe without Russian gas in a mild, reference, and severe winter scenario if the countries act collaboratively. Annual reduction of electricity, industrial gas, and heat demand from November 1, 2022, until October 31, 2023. Countries with involuntarily reduced demand are hatched.

Figure 5 shows the necessary reduction of electricity, industrial gas, and heat demands for the three winter scenarios if European countries act collaboratively. High proactive energy savings, e.g., thermostat adjustments and reduced residential electricity consumption, and a mild winter are sufficient to relieve the strain on the energy system so that the final energy demands must only be reduced to a negligible degree in a few countries. Furthermore, high energy savings would reduce the utilization of coal and lignite power plants by about 30% with respect to the “winter as usual” scenario, with the corresponding environmental benefits.

In the case of a severe winter, the final energy demands are more strongly reduced than in a usual winter. Heat demand is involuntarily reduced in several countries where heat demand is already high in winter with few alternative technologies available. Eastern and Southeastern European countries must use more natural gas to meet their heat demand, hence reducing gas availability for the industrial and electricity sectors. Because essential cross-border capacities are fully utilized, little additional gas can be imported into Southeastern Europe, and most of the region is forced to reduce its electricity and industrial gas demand involuntarily (Figure 2). The situation is exacerbated for countries that are highly dependent on gas in their electricity generation, e.g., Italy.

The results underline the effectiveness and necessity of proactive energy savings to mitigate the impact of the Russian gas shortage. If Europe experiences a mild winter and continues to reduce its energy consumption in preparation for the winter, the energy crisis can be overcome with no major disruptions in energy services and with minimized unwanted environmental impacts. In contrast, a severe winter with higher heat demands exacerbates the challenges of the gas shortage, with high involuntary demand reductions likely because of internal bottlenecks in the European gas flow network.

### Filling gas storage in preparation for next winter

Europe will need to prepare for the following heating season with significantly less gas available to fill the storages over the summer. Experimental procedure S17 investigates different strategies to operate the gas storages and their impact on the necessary demand reductions. If Europe fully discharged the storages and used the gas to avoid any energy demand reduction over the course of the year, the overall demand reduction would be 40% higher during the following heating season.

Although it is highly unlikely that Europe would fully discharge their storages, the results show that overcoming the energy crisis does not only include focusing on the winter of 2022 but also on the winter of 2023. Without Russian gas available, Europe must be careful not to discharge the gas storages to historic levels during the coming heating season, because the gas import availability during the summer is not sufficient to then prepare the storages for the following heating season. Hence, it is essential to ensure foresighted strategies to overcome the energy crisis that can be sustained for years to come.

## Discussion

### Selfish behavior exacerbates the impact of energy crisis for Southeastern Europe

In this study, we provide pathways through potential energy crises by assessing mitigation strategies along the entire gas supply chain in case the European countries behave collaboratively or selfishly. In particular, we investigate how much electricity, heat, and industrial gas demands must be reduced as a consequence of a gas shortage. We make a strong distinction between voluntary and involuntary demand reductions. It is an entirely different implication of how strongly the gas shortage will affect private citizens and the economy whether we voluntarily accept some level of discomfort or if we cannot continue to heat our homes or operate our businesses.

Our results suggest that Europe can get through the winter without significant involuntary demand reductions if the European countries act in solidarity and collaborate. This means voluntarily reducing their own domestic energy demands to supply others with gas they direly need. Our novel formulation to model selfish behavior in centrally planned optimization models allows for quantifying the impact of a lack of collaboration between countries. Although some Central European countries, such as Germany, Belgium, and the Netherlands, profit from avoiding voluntary demand reductions, this selfish behavior could exacerbate the struggle of many other countries, especially in Southeastern Europe. We strongly urge European policymakers to support the sharing of gas among all countries, even if this means voluntarily reducing some of their domestic energy demand because collaboration is the least harmful way to overcome the energy crisis.

### Proactive energy savings are the most cost-effective strategy to overcome the energy crisis

We highlight the importance of proactive energy savings as this has the potential to relieve the European energy system from any additional demand reductions. The amount of energy that we manage to proactively save will determine the extent to which we have to rely on luck regarding the severity of winter. Thus, we call on policymakers to incentivize private citizens and companies alike to reduce their gas consumption in a controlled way wherever possible, even when there is no immediate gas shortage.

In the coming winters, Europe could potentially be in a worse situation than it was in the winter 2022, without a summer with Russian gas available to fill gas storages. It is unlikely that Russian gas imports will return in the summer of 2023, thus Europe must prepare for the next heating season with significantly less gas to fill the storages. Foresighted planning for upcoming winters is essential to overcome future energy crises.

### Diversifying gas imports can largely substitute Russian imports but leads to bottlenecks in the gas network

Besides focusing on where and when the final energy demands can be reduced most effectively, we investigate the optimal supply and transport of gas in the European gas flow network. We find that the optimal compensation strategy for the missing Russian gas imports is a combination of (1) increased non-Russian gas imports (42%), (2) a shift of electricity and heat generation to non-gas-based technologies (28%), and (3) demand reductions as a last resort (30%).

Shifting from Russian gas to importing pipeline gas and LNG from other entry points changes the direction of gas flow in Europe. However, the European gas infrastructure is not designed for this shift, resulting in many vital cross-border capacities being operated at the limit. These bottlenecks can be relieved by additional investments in the gas infrastructure. LNG can help to mitigate the gas shortage even in the short term. Europe appears to have secured significant additional LNG imports and our results show that the gas infrastructure can in fact accept large additional quantities. However, if there is one lesson Europe should learn from the energy crisis, then that being dependent on a foreign country for energy supply is dangerous. Shifting the dependence from Russia to other countries might only postpone the discussion. We recommend harnessing the current movement to intensify the investment in domestic energy supply and in renewable energy technologies.

### Intensified coal-based electricity generation allows supplying demand but increases carbon emissions

Shifting the electricity generation mix to non-gas-based technologies, mainly hard coal and lignite, can reduce the utilization of natural gas turbines by around 30% and thus compensate for 15% of Russian gas imports. Many countries such as Germany and Poland have ample additional coal power capacities, even when taking into account that many units are currently non-operational and cannot be brought back in a reasonable time. We show that by shifting the electricity generation to mainly coal, the electricity demand of almost all countries can be fully supplied in the energy crisis under collaborative action. However, the increased consumption of hard coal and lignite increases the carbon emissions of the electricity sector by 5%, which thwarts Europe’s ability to achieve its climate targets. The total carbon emissions of the entire energy system remain constant with respect to when Russian gas is available, albeit significantly less final energy demand is supplied. Focusing exclusively on the short-term energy crisis while neglecting the long-term decarbonization strategy will probably make us miss Europe’s climate goals.

### Limitations of the study

This analysis focuses on the technical feasibility of mitigating the gas shortage and does not represent the actual socio-economic decision-making in times of crisis. This leads to several limitations that influence the adaption strategy and thus our presented recommendations.

First, we model the adaption strategy as a cost-minimization problem. Although the value of lost load in the industrial sector can be assessed monetarily to a certain degree,^21^^,^^22^ the cost of voluntarily or involuntarily reducing energy consumption in the residential sector is intangible. Moreover, we acknowledge that the limits of voluntary demand reductions are difficult to measure. However, changing the cost for voluntary and involuntary reductions will not change the results in any way if the order of reductions is maintained because demand reductions are always the last resort. There is no fixed order in which the demand in the different sectors will be reduced, only heuristic guidelines that our model tries to incorporate. The sensitivity of the demand reduction cost and maximum shares is qualitatively assessed in Table S11. A rigorous quantitative sensitivity analysis of the parameters may be beneficial. Furthermore, we do not model the economic feedback loop of the gas shortage which has led to increased prices for all energy carriers. The elasticity of the electricity, heat, and gas demand is neglected in this study.

Second, the study does not attempt to depict and incorporate political decisions that were or are made during this crisis. As examples, these include Germany’s decision to phase out electricity generation from nuclear in April 2023,^30^ or Hungary’s decision to intensify its ties to Russia and expand Russian gas imports.^31^ We provide a conservative scenario by assuming a complete disruption of Russian gas, which is currently not the case in Europe, while preventing additional technology investments. Furthermore, the consideration of long-term climate goals in the current situation may lead to cost-suboptimal decisions in the short term. One example is the hesitation by many countries to increase the utilization of coal power plants. We support and commend these efforts; however, they are out of the scope of this analysis.

Finally, the current energy crisis impacts all participants in the energy system, from large industrial gas consumers to individual citizens. Predicting optimal mitigation strategies through a single techno-economic optimization model neglects the suboptimality and heterogeneity of real-world decision-making. The trade-offs (1) between the sectors of the energy system and (2) between countries are in reality not optimally solved.

## STAR★Methods

### Key resources table

**Table:** 

REAGENT or RESOURCE	SOURCE	IDENTIFIER
**Deposited data**		

Original data to reproduce figures and results	This paper	Zenodo: https://doi.org/10.5281/zenodo.7554923

**Software and algorithms**		

Original code to reproduce figures and results	This paper	Zenodo: https://doi.org/10.5281/zenodo.7554923

### Resource availability

#### Lead contact

Further information and requests for resources should be directed to and will be fulfilled by the lead contact, Giovanni Sansavini (sansavig@ethz.ch).

#### Materials availability

No materials were used in this study.

### Method details

The detailed mathematical description of the optimization model, input data, and assumptions are presented in the Experimental procedures S1–S16 and Tables S1–S10.

#### Operational optimization model

We investigate the impact of a complete Russian gas disruption on the European energy system by optimizing the operation of existing energy conversion, transport, and storage technologies in 28 European countries (EU27 with CH, NO, UK, without CY, MT). The countries are resolved at NUTS0 level (country level) with edges connecting the centroids. We solve a linear optimization problem with hourly resolved input data for an entire year, from November 1, 2022, until October 31, 2023.

The objective of the optimization is to supply the exogenous and inelastic demands of electricity, heat, and industrial gas at the lowest cost for the entire energy system. The final energy demands are supplied by various conversion technologies in each sector (Table S1). The electricity and heat demands are provided as hourly time series, whereas the industrial gas demand is assumed to be constant throughout the year. In our analysis, no alternative is available to replace industrial gas, which is therefore the most vulnerable sector to reduced supplies from neighboring countries. Reduced industrial gas consumption does not necessarily mean that the industrial output is reduced proportionally. Some industries can switch from gas to other feedstocks. However, experts suggest that the fuel-switch potential is limited and energy efficiency enhancements are largely exhausted in industry.^21^

Only the two energy carriers gas and electricity can be transported and stored. Gas is transported via natural gas pipelines and stored in natural gas storages; electricity is transported via power lines and stored in pumped hydro power plants. We apply a periodicity constraint for the storage technologies to enforce that the storage levels at the beginning and at the end of the year match.

We aggregate the input time series to 1400 representative time steps, using the time series aggregation package tsam 2.1.0.^32^ The optimization model is formulated in Pyomo 6.4.2^33^ with Python 3.9.13 and solved with Gurobi 9.5.2^34^ on an AMD Ryzen Threadripper 3960X 24-core processor, 3.79 GHz machine with 128 GB RAM.

#### Voluntary and involuntary demand reductions

Demand reductions $R_{c,n,t}$ for all energy carriers c∈C, nodes n∈N, and time steps t∈T are modeled as slack variables that are penalized in the objective function. Demand reductions are always more expensive than supplying the energy demand by operating the technologies. Hence, the optimizer tries to avoid reducing the final energy demands. We assume that the demand can be reduced on two levels. The first one, $R_{c,n,t}^{V}$, describes the voluntary demand reduction, which is penalized at a lower cost, $k_{c}^{V}$; the second one, $R_{c,n,t}^{I}$, describes the involuntary demand reduction, which is penalized at a higher cost, $k_{c}^{I}>k_{c}^{V}$. Since $k_{c}^{I}>k_{c}^{V}$, the optimizer will first use $R_{c,n,t}^{V}$ until $R_{c,n,t}^{V}=s_{c}^{V}D_{c,n,t}$, before it uses $R_{c,n,t}^{I}$. The total demand reduction $R_{c,n,t}$ is the sum of $R_{c,n,t}^{V}$ and $R_{c,n,t}^{I}$. Both $R_{c,n,t}^{V}$ and $R_{c,n,t}^{I}$ are limited to a share ($s_{c}^{V}$ and $s_{c}^{I}$, respectively) of the demand $D_{c,n,t}$:

(Equation 1) $$R_{c,n,t}^{V}\leq s_{c}^{V}D_{c,n,t}\forall c\in C,\forall n\in N,\forall t\in T\text{,}$$

(Equation 2) $$R_{c,n,t}^{I}\leq s_{c}^{I}D_{c,n,t}\forall c\in C,\forall n\in N,\forall t\in T\text{.}$$

Below Table lists the order, cost $k_{c}$, and maximum share $s_{c}$ of reducing the final energy demands. The cost of demand reductions enforces the order in which the demands are reduced. We assume that it is preferable to turn down heating than to reduce the industrial output voluntarily, followed by the voluntary electricity demand reduction. Electricity and industrial gas demand are involuntarily reduced at around the same point. Afterwards, the heat demand is the last to be reduced involuntarily.

**Table:** Order, cost, and maximum share of reducing final energy demands voluntarily (rows 1–3) and involuntarily (rows 4–6)

Order	Energy sector	Reduction level	$k_{c}$ [Euro/MWh]	$s_{c}$ [%]
1 (first)	Heat	voluntary	200^g^	5.73
2	Industrial gas	voluntary	250^a^	15
3	Electricity	voluntary	1000^g^	3.165
4	Industrial gas	involuntary	1648^b^	72^c^
5	Electricity	involuntary	3000^d^	71.235^e^
6 (last)	Heat	involuntary	2000^g^	56.77^f^

The cost of reducing electricity and heat must be multiplied by 0.55 (efficiency of combined cycle natural gas turbines) and 0.93 (efficiency of natural gas boilers), respectively, to estimate the cost of reducing gas consumption in the electricity and heat sector.

a Average voluntary reimbursement in industrial sector.^21^

b Economic value-added intensity of gas-intensive industry.^22^

c Share of gas consumption of the gas-intensive industry (87%) minus voluntary reduction limit.^22^

d Maximum price limit for European Power Futures in 2021.^23^

e Share of non-domestic electricity consumption in EU27 (74.4%) minus voluntary reduction limit.^4^

f Share of non-domestic heat consumption in EU27 (62.5%) minus voluntary reduction limit.^4^

g Assumption.

The order, the demand reduction price, and the maximum share of reduced demand are based on educated, personal assessment since no prioritization is published by official sources. We follow the regulation by the EU^20^ to voluntarily reduce gas consumption by 15%. The limit of voluntary demand reductions assumes that the 15% reduction of gas consumption could not be substituted by other technologies but would result in a direct reduction of the final energy demand. Applied to each sector, we multiply the limit with the share of natural gas consumption in electricity (21.1%) and heat generation (38.2%) in Europe in 2020,^35^ which results in reduction limits of 3.165% and 5.73%, respectively. The sensitivity of the parameters is qualitatively assessed in Table S11.

#### Selfish decision-making

The optimization model is formulated as a centrally planned model, where the total operational cost is minimized for the entire energy system. Central planning inherently leads to collaborative behavior among countries. Specifically, it is more beneficial for the entire system to reduce demand voluntarily in one country to keep other countries from reducing demand involuntarily since this is significantly more expensive.

Our definition of selfish behavior is that countries will first attempt to supply their entire final energy demands before they share any excess gas and electricity with neighboring countries. Hence, countries will not support other countries by voluntarily reducing their domestic energy demands and exporting gas and electricity. We model the selfish behavior of countries by implementing a binary decision between voluntary demand reductions and energy exports. In this model, transportable energy carriers $c\in C^{T}\subset C$ are gas and electricity. If a selfish country n∈N decides to share gas or electricity with other countries $n^{\prime }\in N^{\prime }=\left\{n^{\prime }\in N,|,n^{\prime }\neq n\right\}$, it cannot reduce its demand of the associated energy carriers voluntarily. Thus under selfish decision-making, if a country shares gas or electricity, all domestic demand reductions of associated carriers become involuntary. Vice versa, if the demand is reduced voluntarily, the transported flows to all other countries are zero. Imports from other countries are not impacted. Here, associated carriers $c^{\prime }\in C_{c}^{\prime }$ are those energy carriers that gas and electricity can be converted into. For gas, associated carriers are electricity, heat, and industrial gas. For electricity, this is heat and electricity itself.

In our model, the binary decision is implemented as a generalized disjunctive programming (GDP) formulation. GDP sets logical relationships between groups of constraints, called a disjunction, where *M* disjuncts $d^{m}$ form OR-relationships^36^:

(Equation 3) $$d^{1}\vee d^{2}\vee \cdots \vee d^{M}\text{.}$$

The selfish decision-making is a disjunction of M=2 disjuncts: The first disjunct constrains the annual transport flow $F_{c,n,n^{\prime },t}$ for all transportable energy carriers $c\in C^{T}$ for all countries n∈N to all other nodes $n^{\prime }\in N^{\prime }$:

(Equation 4) $$d^{1}:\left[\sum_{t\in T},\sum_{n^{\prime }\in N^{\prime }},F_{c,n,n^{\prime },t}\leq \varepsilon \right]\forall c\in C^{T},n\in N\text{,}$$

with the time steps t∈T. Note that we slightly relax the complementarity constraints to $\varepsilon =10^{-3}$ to reduce the computational burden.

The second disjunct constrains the annual voluntary demand reduction $R_{c^{\prime },n,t}^{V}$ for all associated energy carriers $c^{\prime }\in C_{c}^{\prime }$:

(Equation 5) $$d^{2}:\left[\sum_{t\in T},\sum_{c^{\prime }\in C_{c}^{\prime }},R_{c^{\prime },n,t}^{V}\leq \varepsilon \right]\forall c\in C^{T},n\in N\text{.}$$

The disjunction for all $c\in C^{T}$ and n∈N is then:

(Equation 6) $$\left[\sum_{t\in T}\sum_{n^{\prime }\in N^{\prime }}F_{c,n,n^{\prime },t}\leq \varepsilon \right]\vee \left[\sum_{t\in T}\sum_{c^{\prime }\in C_{c}^{\prime }}R_{c^{\prime },n,t}^{V}\leq \varepsilon \right]\forall c\in C^{T},n\in N\text{.}$$

The disjunction is reformulated internally in Pyomo to binary big-M constraints.^36^ The upper bounds for $F_{c,n,n^{\prime },t}$ are set to the existing capacity of the transport technologies $C_{j,n,n^{\prime }}$. Since a transport technology j∈J can only transport one energy carrier, the relationship j=j(c) is unique. The upper bounds for $R_{c^{\prime },n,t}^{V}$ are set to the demand $D_{c^{\prime },n,t}$.

## Data Availability

• The data needed to reproduce all figures and results have been deposited at Zenodo and are publicly available as of the date of publication. The DOI is listed in the key resources table. • All original code has been deposited at Zenodo and is publicly available as of the date of publication. DOIs are listed in the key resources table. • Any additional information required to reanalyze the data reported in this paper is available from the lead contact upon request.

## Supplemental information

Document S1. Experimental procedure S1–17, Figures S1 and S2, and Tables S1–S11
