# Supplementary material for: Collaborative and selfish mitigation strategies to tackle energy scarcity: The case of the European gas crisis
Source: iScience. 2023 Apr 27;26(5):106750. doi: 10.1016/j.isci.2023.106750 (PMC10214717; doi:10.1016/j.isci.2023.106750)
Supplement: Document S1. Experimental procedure S1–17, Figures S1 and S2, and Tables S1–S11 [file mmc1.pdf]

**iScience, Volume *26***

## Supplemental information

# Collaborative and selfish mitigation strategies to tackle energy scarcity: The case of the European gas crisis

**Jacob Mannhardt, Paolo Gabrielli, and Giovanni Sansavini**

## Supplemental items

### *Table S1: Modeled conversion technologies*

Table S1: Conversion technologies in optimization model with respective output and input carrier, and conversion efficiency. Technologies of the natural gas sector in italic. The capacities of technologies are rated by their output carrier. CCGT: combined-cycle gas turbine. Related to STAR Methods.

| Conversion technology | Output carrier | Input carrier | Efficiency |
|---|---|---|---|
| *Natural gas turbine (CCGT)* | Electricity | *Natural gas* | 0.560[39] |
| Hard coal plant | | Hard coal | 0.454[39] |
| Lignite coal plant | | Lignite | 0.427[39] |
| Nuclear power plant | | Uranium | 0.333[39] |
| Oil power plant | | Oil | 0.467[39] |
| Waste power plant | | Waste | 0.371[39] |
| Biomass power plant | | Biomass | 0.371[39] |
| Onshore wind | | | |
| Offshore wind | | | |
| Solar photovoltaic | | | |
| Run-of-river hydropower | | | |
| Reservoir hydropower | | | |
| *Natural gas boiler* | Heat | *Natural gas* | 0.93[33] |
| Hard coal boiler | | Hard coal | 0.93[33] |
| Oil boiler | | Oil | 0.93[33] |
| Waste boiler | | Waste | 0.80[5] |
| Biomass boiler | | Biomass | 0.85[5] |
| Heat pump | | Electricity | 3.70[33] |
| Electric boiler | | Electricity | 0.99[33] |
| *LNG terminal* | *Natural gas* | LNG | 1 |
| Industrial gas consumer | Industrial gas | *Natural gas* | 1 |

*Table S2: Modeled transport and storage technologies*

Table S2: Transport and storage technologies in optimization model with reference carrier. Relative loss of transport technologies is per distance and transported energy. For the storage technologies, we assume equal discharging and charging efficiencies and no self-discharge[58]. Related to STAR Methods.

| **Transport technology** | **Reference carrier** | **Relative loss [1/km]** |
|---|---|---|
| Power line | Electricity | 0.00005[57] |
| Natural gas pipeline | Natural gas | 0.00005[1] |

| **Storage technology** | **Reference carrier** | **Round-trip efficiency** |
|---|---|---|
| Pumped hydro | Electricity | 0.780[50] |
| Natural gas storage | Natural gas | 0.995[2] |

[1] Assumption; value for power line[57]
[2] Assumption to avoid simultaneous charging and discharging.

*Table S3: Additional LNG import capacities as reaction to gas shortage*

Table S3: Additional hourly LNG import capacities as reaction to gas shortage. If the start year of additional capacity is 2021 or 2022, then the capacities are available from beginning of optimization. If the start year of additional capacity is 2023, then the capacities available from January 1, 2023. Related to STAR Methods.

| **Country** | **Capacity [GW]** | **Start year** |
|---|---|---|
| DE | 3.9022 [4] | 2023 |
| EE | 2.917 [13] | 2023 |
| FI | 0.2 [41] | 2022 |
| FI | 2.917 [13] | 2023 |
| PL | 6.914 [32] | 2022 |
| HR | 2.8996 [47] | 2021 |

*Table S4: Additional cross-border gas pipeline capacities as reaction to gas shortage*

Table S4: Additional cross-border gas pipeline capacities as a reaction to gas shortage from[20]. Related to STAR Methods.

| Connection | New/additional capacity [GW] |
|---|---|
| DK-PL | 11.15 |
| EE-FI | 2.93 |
| EL-BG | 3.35 |
| EL-IT | 20.30 |
| FI-EE | 2.93 |
| FR-DE | 1.29 |
| NO-DK | 11.15 |
| PL-DK | 3.35 |

*Table S5: Hourly pipeline gas export capacities to Europe*

Table S5: Hourly pipeline export capacities of exporting regions to Europe. Base availability applies to scenario with Russian gas available. Obtained from [16]; the capacity value is selected as min, mean, or max (as indicated) of provided range to resemble historic values by [30]. Additional availability under gas shortage from [30]. Converted from bcm to GW. Related to STAR Methods.

| Exporting region | Base availability [GW] | Add. availability gas shortage [GW] | Connected countries |
|---|---|---|---|
| Algeria | 36.53 (max) | 2.23 | ES, IT |
| Libya | 5.36 (mean) | 0 | ES, IT |
| Turkey | 1.34 (mean) | 0 | EL |
| Azerbaijan | 7.43 (min) | 3.18 | EL |
| Turkmenistan | 0 (min) | 0 | EL |
| Russia | 182.27 (mean) | 0 | BG, RO, HU, SK, PL, LT, EE, DE, FI |
| Norway | 141.63 (max) | 2.23 | NO |

*Table S6: Yearly LNG gas export capacities to Europe*

Table S6: Yearly LNG export capacities of exporting regions to Europe. Base availability applies to the scenario with Russian gas available. Obtained from[16]; the capacity value is selected as min, mean, or max (as indicated) of provided range to resemble historic values by[30]. Base availability of non-Russian LNG is selected at 25% of the provided range (mean of range between min and mean value, mean[min,mean]). Additional availability under gas shortage from[30]. Countries in parentheses are only available in the scenario without Russian gas available. Converted from bcm to GWh. Related to STAR Methods.

| Exporting region | Base availability [GWh] | Add. availability gas shortage [GWh] | Connected countries |
|---|---|---|---|
| non-Russian LNG | 722,218 (mean[min,mean]) | 537,319 | BE, EL, ES, FR, HR, IT, LT, NL, PT, UK, (DE, EE, FI, PL) |
| Russian LNG | 23,232 (mean) | 0 | BE, EL, ES, FR, HR, IT, LT, NL, PT, UK, (DE, EE, FI, PL) |

*Table S7: Existing capacity of heat generation technologies*

Table S7: Existing capacities of heat generation technologies for all 28 countries. Calculated with the methodology described in Procedure S13. Related to STAR Methods.

| Country | Natural gas boiler [GW] | Hard coal boiler [GW] | Oil boiler [GW] | Waste boiler [GW] | Biomass boiler [GW] | Heat pump [GW] | Electric boiler [GW] |
|---|---|---|---|---|---|---|---|
| AT | 8.33 | 0.20 | 4.82 | 0.41 | 8.87 | 2.96 | 2.63 |
| BE | 20.41 | 0.18 | 14.29 | 0.11 | 2.15 | 2.85 | 1.72 |
| BG | 2.19 | 0.98 | 0.73 | 0.01 | 4.20 | 0.83 | 2.40 |
| CH | 11.29 | 0.27 | 6.54 | 0.56 | 12.02 | 4.02 | 3.57 |
| CY | 0.00 | 0.00 | 0.09 | 0.00 | 0.01 | 0.06 | 0.04 |
| CZ | 12.90 | 5.35 | 1.58 | 0.17 | 7.85 | 2.25 | 3.38 |
| DE | 132.84 | 5.98 | 68.78 | 2.10 | 32.28 | 9.16 | 14.64 |
| DK | 5.26 | 0.81 | 2.98 | 1.49 | 10.61 | 2.21 | 0.52 |
| EE | 0.86 | 0.08 | 0.46 | 0.05 | 2.63 | 0.06 | 0.37 |
| EL | 3.73 | 0.35 | 11.10 | 0.00 | 3.73 | 6.42 | 2.64 |
| ES | 14.53 | 0.35 | 15.78 | 0.01 | 5.02 | 3.57 | 3.42 |
| FI | 1.27 | 1.90 | 3.23 | 0.29 | 6.78 | 5.21 | 2.31 |
| FR | 62.40 | 0.44 | 36.81 | 1.18 | 24.28 | 18.93 | 28.01 |
| HR | 3.54 | 0.01 | 1.48 | 0.00 | 4.16 | 0.68 | 0.81 |
| HU | 20.26 | 0.34 | 2.28 | 0.09 | 5.24 | 0.75 | 2.18 |
| IE | 3.15 | 1.13 | 5.67 | 0.00 | 0.14 | 0.57 | 1.15 |
| IT | 103.51 | 0.02 | 20.96 | 0.15 | 25.19 | 11.26 | 9.65 |
| LT | 1.57 | 0.25 | 0.51 | 0.12 | 3.78 | 0.16 | 0.18 |
| LU | 1.24 | 0.00 | 0.93 | 0.00 | 0.37 | 0.26 | 0.14 |
| LV | 1.58 | 0.01 | 0.83 | 0.00 | 2.65 | 0.08 | 0.27 |
| MT | 0.00 | 0.00 | 0.01 | 0.00 | 0.00 | 0.00 | 0.02 |
| NL | 39.96 | 0.05 | 3.35 | 0.27 | 2.64 | 1.71 | 1.99 |
| NO | 0.15 | 0.03 | 2.43 | 0.52 | 2.09 | 11.58 | 4.84 |
| PL | 19.81 | 35.34 | 11.87 | 0.37 | 18.67 | 1.67 | 2.03 |
| PT | 0.59 | 0.00 | 1.11 | 0.00 | 0.79 | 0.24 | 0.62 |
| RO | 7.35 | 0.32 | 1.64 | 0.00 | 5.01 | 0.07 | 0.10 |
| SE | 0.67 | 0.08 | 1.78 | 1.64 | 8.02 | 9.19 | 3.84 |
| SI | 0.73 | 0.26 | 1.28 | 0.01 | 1.66 | 0.52 | 0.54 |
| SK | 5.05 | 0.68 | 0.22 | 0.04 | 1.90 | 0.59 | 0.80 |
| UK | 120.14 | 1.97 | 22.39 | 0.16 | 7.77 | 6.74 | 11.37 |

### *Table S8: Prices and carbon intensities of energy carriers*

Table S8: Prices (yearly average) and carbon intensities of energy carriers that can be imported. We assume country- and time-dependent prices. Waste is treated as an unavoidable byproduct and thus does not have a price and carbon intensity. This aligns with the EU Emission Trading System (EU ETS) where waste will not be included until 2026[27]. Related to STAR Methods.

| **Energy carrier** | **Fuel price [Euro/MWh]** | **Carbon intensity [tons/MWh]** |
|---|---|---|
| Natural gas | 12.95 . . . 27.02[23] | 0.202[39] |
| LNG | 17.53 . . . 25.25[23] | 0.202[39] |
| Hard coal | 9.56[7,39] | 0.341[39] |
| Lignite | 3.96[7,39] | 0.364[39] |
| Uranium | 2.87[39] | 0[39] |
| Oil | 24.15[21] | 0.279[39] |
| Waste | 0 | 0 |
| Biomass | 13.68[34] | 0 |

*Table S9: Variable operational costs*

Table S9: Variable operational costs (OPEX) of electricity generation technologies (without fuel costs) and LNG terminals as assumed in this study. We assume no variable operational costs for heat generation technologies and industrial gas consumers. Related to STAR Methods.

| **Conversion technology** | **Variable OPEX [Euro/MWh]** |
|---|---|
| Natural gas turbine (CCGT) | 2.14 [39] |
| Hard coal plant | 3.89 [39] |
| Lignite coal plant | 4.24 [39] |
| Nuclear power plant | 8.44 [39] |
| Oil power plant | 5.64 [39] |
| Waste power plant | 6.21 [39] |
| Biomass power plant | 6.21 [39] |
| Onshore wind | 0 [39] |
| Offshore wind | 2.14 [39] |
| Solar photovoltaic | 0 [39] |
| Run-of-river hydropower | 0 [39] |
| Reservoir hydropower | 0 [39] |
| LNG terminal | 2.48 [11,54] |

*Table S10: Eurostat codes*

Table S10: Used Eurostat energy balances (nrg_bal) and energy carrier (siec) in datasets. Related to STAR Methods.

| Code | Label | Type | Dataset |
|---|---|---|---|
| NRGSUP | Total Supply | nrg_bal | nrg_bal_c |
| PPRD | Primary Production | nrg_bal | nrg_bal_c |
| IMP | Imports | nrg_bal | nrg_bal_c |
| GHP | Gross Heat Production | nrg_bal | nrg_bal_c |
| TI_EHG_E | Transformation input - electricity and heat generation - energy use | nrg_bal | nrg_bal_c |
| FC_OTH_E | Final consumption - other sectors energy use | nrg_bal | nrg_bal_c |
| FC_OTH_HH_E | Final consumption - other sectors households - energy use | nrg_bal | nrg_bal_c |
| FC_OTH_CP_E | Final consumption - other sectors commercial and public services energy use | nrg_bal | nrg_bal_c |
| FC_OTH_HH_E | Final consumption - other sectors households - energy use | nrg_bal | nrg_d_hh |
| FC_OTH_HH_E_LE | Final consumption - other sectors households - energy use lighting and electrical appliances | nrg_bal | nrg_d_hh |
| FC_OTH_HH_E_CK | Final consumption - other sectors households - energy use - cooking | nrg_bal | nrg_d_hh |
| G3000 | Natural gas | siec | nrg_bal_c |
| P1000 | Peat and peat products | siec | nrg_bal_c |
| O4000XBIO | Oil and petroleum products | siec | nrg_bal_c |
| R5110-5150_W6000RI | Primary solid biofuels | siec | nrg_bal_c |
| R5300 | Biogases | siec | nrg_bal_c |
| E7000 | Electricity | siec | nrg_bal_c |
| H8000 | Heat | siec | nrg_bal_c |
| W6100_6220 | Non-renewable waste | siec | nrg_bal_c |
| C0350-0370 | Manufactured gases | siec | nrg_bal_c |
| N900H | Nuclear heat | siec | nrg_bal_c |
| C0000X0350-0370 | Solid fossil fuels | siec | nrg_bal_c |

*Table S11: Qualitative sensitivity assessment of demand reduction costs and shares*

Table S11: Qualitative sensitivity assessment of the impact of a change in the demand reduction costs $k_c^{\mathrm{V}}$ and $k_c^{\mathrm{I}}$, and maximum demand reduction shares $s_c^{\mathrm{V}}$ and $s_c^{\mathrm{I}}$. Related to STAR Methods.

| Parameter change | | Impact on results |
|---|---|---|
| All $k_c^{\mathrm{I}}$ increase equally | $\rightarrow$ | None |
| All $k_c^{\mathrm{I}}$ decrease equally but $> k_c^{\mathrm{V}}$ | $\rightarrow$ | None |
| One $k_c^{\mathrm{I}}$ increases/decreases but order retained | $\rightarrow$ | None |
| One $k_c^{\mathrm{I}}$ increases/decreases but order changes | $\rightarrow$ | Increased/decreased $R_c^{\mathrm{I}}$ of energy sector that climbed/fell in the reduction order |
| One/all $s_c^{\mathrm{I}}$ increases/decreases | $\rightarrow$ | Minor impact because limit rarely reached. Decreases/increases $R_c^{\mathrm{I}}$ in other energy sectors |
| All $k_c^{\mathrm{V}}$ increase equally but $< k_c^{\mathrm{I}}$ | $\rightarrow$ | None |
| All $k_c^{\mathrm{V}}$ decrease equally but higher than marginal cost of production | $\rightarrow$ | None |
| All $k_c^{\mathrm{V}}$ decrease equally but lower than marginal cost of production | $\rightarrow$ | Increased $R_c^{\mathrm{V}}$. Unrealistic, because demand reduction is last resort |
| One $k_c^{\mathrm{V}}$ increases/decreases but order retained | $\rightarrow$ | None |
| One $k_c^{\mathrm{V}}$ increases/decreases but order changes | $\rightarrow$ | Increased/decreased $R_c^{\mathrm{V}}$ of energy sector that climbed/fell in the reduction order |
| One/all $s_c^{\mathrm{V}}$ increases/decreases | $\rightarrow$ | Decreases/increases $R_c^{\mathrm{V}}$ in other energy sectors. Significantly decreases/increases $R_c^{\mathrm{I}}$ in all energy sectors. |
| Regional impacts (hypothetical): | | |
| $k_c^{\mathrm{V}}/k_c^{\mathrm{I}}$ increases/decreases for one country but order changes | $\rightarrow$ | Increased/decreased $R_c^{\mathrm{V}}/R_c^{\mathrm{I}}$ of energy sector that climbed/fell in the reduction order. Energy flows to country are increased/reduced |
| $s_c^{\mathrm{V}}/s_c^{\mathrm{I}}$ increases/decreases for one country | $\rightarrow$ | More/less available energy for other countries. Higher/lower demand reduction in this country |

*Figure S1: Hourly reduction of final energy demands*

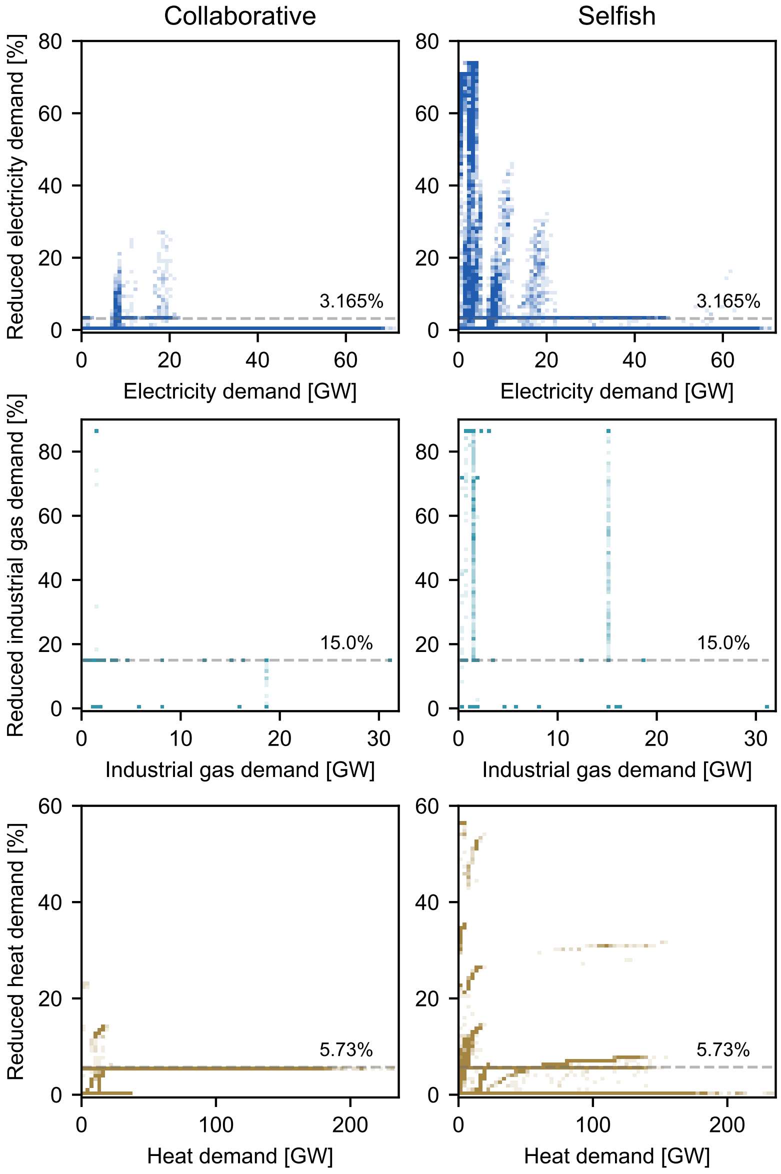


Figure S1: Hourly reduction of electricity, industrial gas, and heat demands for all countries in Europe if the countries collaborate (left) or if the countries act selfishly (right) from November 1, 2022, and October 31, 2023. The limit between voluntary and involuntary demand reduction is indicated by dashed lines. Related to Fig. 1.

*Figure S2: Gas sources of energy system*

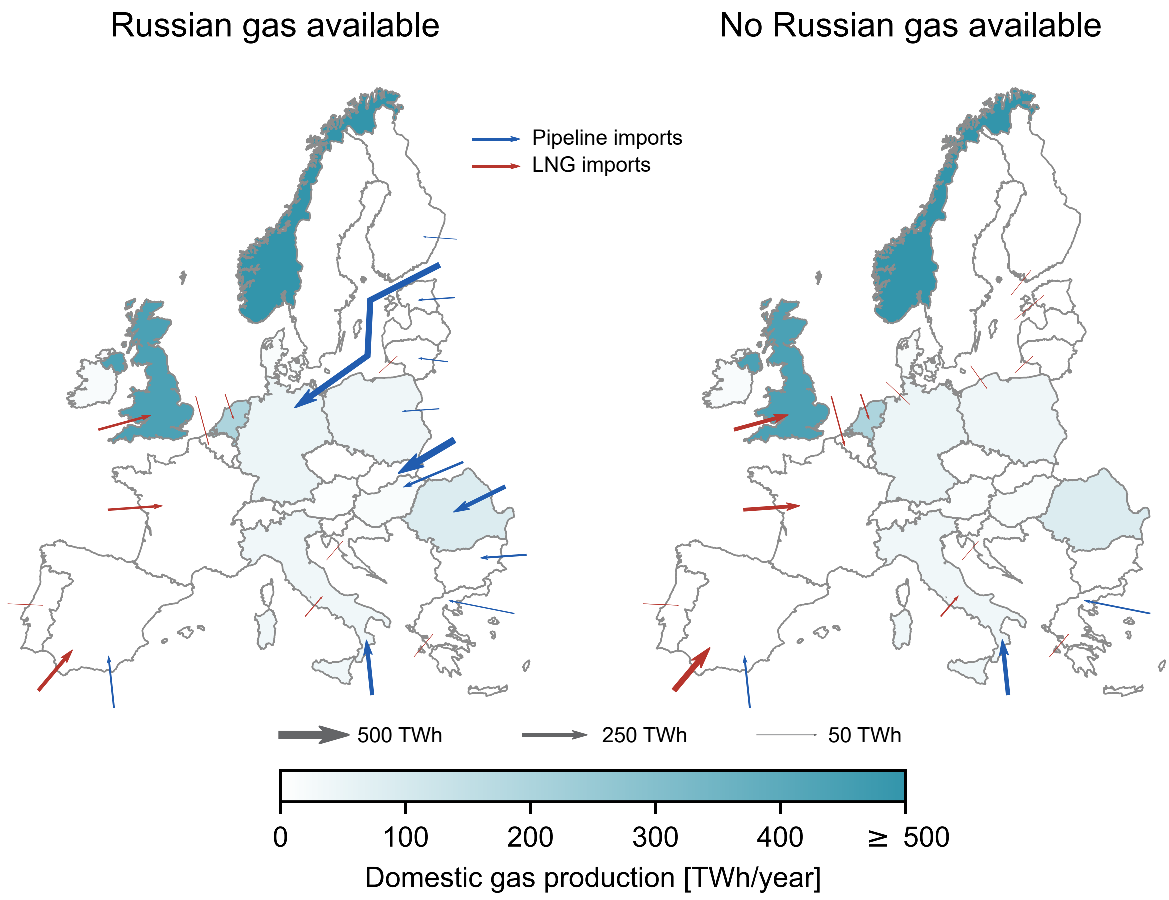


Figure S2: Schematic depiction of gas sources and entry points for the scenarios with and without Russian gas available. Domestic production is shown by color bar (note that Norway produces around 1200 TWh of natural gas annually). Annual pipeline imports are in blue, LNG imports are in red. Related to Fig. 2.

## Supplemental Experimental Procedures

*Procedure S1:. Objective function of operational optimization model*

We investigate the operation of the European energy system in the optimization framework ZEN-garden (Zero-emissions Energy Networks), developed at the Reliability and Risk Engineering laboratory at ETH Zurich. The multipurpose optimization framework ZEN-garden optimizes the design and operation of energy system models to investigate transition pathways towards decarbonization.

The optimization model is formulated as a linear cost-minimization problem, minimizing the total operational cost $K^{\mathrm{tot}}$ for all countries $n \in \mathcal{N}$ for all time steps $t \in \mathcal{T}$. The goal of the optimizer is to supply the final energy demands $D_{c,n,t}$ of carrier $c \in \mathcal{C}$ at the lowest cost by operating conversion technologies $i \in \mathcal{I}$, transport technologies $j \in \mathcal{J}$, and storage technologies $s \in \mathcal{S}$. In this study, each conversion technology has at most one input carrier $c^{\mathrm{i}}$ and one unique reference carrier $c^{\mathrm{r}}$, i.e, the output carrier by which the existing capacity $C_{i,n}$ is rated.

The total costs include:

- Fuel costs $K^{\mathrm{F}}_{c,n,t}$ for all energy carriers $c \in \mathcal{C}$ (product of energy carrier price $k_{c,n,t}$ and imported flow of energy carrier from outside the energy system boundaries $F^{\mathrm{imp}}_{c,n,t}$)
- Cost of carbon emissions $K^{\mathrm{C}}_{c,n,t}$ (product of carbon price $k^{\mathrm{C}}$, carbon emission intensity of energy carrier $e_{c,n,t}$, and $F^{\mathrm{imp}}_{c,n,t}$)
- Cost of demand reductions $K^{\mathrm{R}}_{c,n,t}$ (product of demand reduction price $k^{\mathrm{V}}_c$ and $k^{\mathrm{I}}_c$, and reduced demand $R^{\mathrm{V}}_{c,n,t}$ and $R^{\mathrm{I}}_{c,n,t}$, sum of voluntary $(\mathrm{V})$ and involuntary $(\mathrm{I})$ demand reduction costs)
- Variable operational cost $K^{\mathrm{O}}_{i,n,t}$ for $i \in \mathcal{I}$ (product of specific variable operational cost $k^{\mathrm{O}}_{i,n,t}$ and the output flow $F^{\mathrm{o}}_{i,c,n,t}$)

$K^{\mathrm{tot}}$ is formulated as:

$$K^{\mathrm{tot}} = \sum_{n\in\mathcal{N}} \sum_{t\in\mathcal{T}} \left( \sum_{c\in\mathcal{C}} \left( K^{\mathrm{F}}_{c,n,t} + K^{\mathrm{C}}_{c,n,t} + K^{\mathrm{R}}_{c,n,t} \right) + \sum_{i\in\mathcal{I}} K^{\mathrm{O}}_{i,n,t} \right). \tag{1}$$

*Procedure S2:. Energy balance of operational optimization model*

The energy balance ensures that supply and demand match for all $c \in \mathcal{C}$, $n \in \mathcal{N}$, $t \in \mathcal{T}$. The supply side consists of:

- $F^{\mathrm{o}}_{i,c,n,t}$,
- the transported flow $F_{c,n',n,t}$ from all other nodes $n' \in \mathcal{N}'$ with $\mathcal{N}' = \{n' \in \mathcal{N} | n' \neq n\}$ to $n$, minus the losses $F^{\mathrm{l}}_{c,n,n',t}$,
- the discharge flow $F^{\mathrm{d}}_{s,c,n,t}$ from all storage technologies $s \in \mathcal{S}$,
- and $F^{\mathrm{imp}}_{c,n,t}$.

The demand side consists of:

- the input flow $F^{\mathrm{i}}_{i,c,n,t}$,
- the transported flow $F_{c,n,n',t}$ to all other nodes $n' \in \mathcal{N}'$ from $n$,
- the charge flow $F^{\mathrm{c}}_{s,c,n,t}$,
- $D_{c,n,t}$,
- and $R^{\mathrm{V}}_{c,n,t}$ and $R^{\mathrm{I}}_{c,n,t}$.

All flows, demands, and demand reductions are non-negative real numbers $\mathbb{R}_0^+$. Note that exports to outside the energy system boundaries are disabled in this model. The complete energy balance is:

$$\begin{aligned}
&\sum_{i\in\mathcal{I}} \left(F^{\mathrm{o}}_{i,c,n,t} - F^{\mathrm{i}}_{i,c,n,t}\right) + \\
&\sum_{n'\in\mathcal{N}'} \left(F_{c,n',n,t} - F_{c,n,n',t} - F^{\mathrm{l}}_{c,n,n',t}\right) + \\
&\sum_{s\in\mathcal{S}} \left(F^{\mathrm{d}}_{s,c,n,t} - F^{\mathrm{c}}_{s,c,n,t}\right) + \\
&F^{\mathrm{imp}}_{c,n,t} - \left(D_{c,n,t} - (R^{\mathrm{V}}_{c,n,t} + R^{\mathrm{I}}_{c,n,t})\right) \\
&= 0 \qquad \forall c\in\mathcal{C}, \forall n\in\mathcal{N}, \forall t\in\mathcal{T}. \qquad (2)
\end{aligned}$$

$F^{\mathrm{o}}_{i,c,n,t}$ is constrained by the product of the capacity factor $CF_{i,n,t}$ and $C_{i,n}$, if $c = c^{\mathrm{r}}$:

$$F^{\mathrm{o}}_{i,c,n,t} \leq CF_{i,n,t} C_{i,n} \qquad \forall i\in\mathcal{I}, \forall n\in\mathcal{N}, \forall t\in\mathcal{T}. \qquad (3)$$

The conversion efficiency $\eta_i$ is the ratio of $F^{\mathrm{o}}_{i,c^{\mathrm{r}},n,t}$ and $F^{\mathrm{i}}_{i,c^{\mathrm{i}},n,t}$:

$$\eta_i = \frac{F^{\mathrm{o}}_{i,c^{\mathrm{r}},n,t}}{F^{\mathrm{i}}_{i,c^{\mathrm{i}},n,t}} \qquad \forall i\in\mathcal{I}, \forall n\in\mathcal{N}, \forall t\in\mathcal{T}. \qquad (4)$$

This study utilizes an energy transfer model between nodes, as is often deployed in large-scale energy system models[44,57]. In general, simplifying the nonlinear flow equations to an energy transfer formulation yields a satisfactory trade-off between accuracy and computational complexity, in particular on a national spatial resolution[40]. Analogously to the conversion technologies, the flows along the edges $F_{c,n,n',t}$ are constrained by the corresponding transfer capacity $C_{j,n,n'}$ with the unique relationship $c = c(j)$:

$$F_{c,n,n',t} \leq C_{j,n,n'} \qquad \forall j\in\mathcal{J}, \forall n\in\mathcal{N}, \forall n'\in\mathcal{N}', \forall t\in\mathcal{T}. \qquad (5)$$

The flow loss $F^{\mathrm{l}}_{c,n,n',t}$ is the product of $F_{c,n,n',t}$, the distance $l_{n,n'}$ and the relative loss coefficient $\rho_j$:

$$F^{\mathrm{l}}_{c,n,n',t} = F_{c,n,n',t} l_{n,n'} \rho_j \qquad \forall j\in\mathcal{J}, \forall n\in\mathcal{N}, \forall n'\in\mathcal{N}', \forall t\in\mathcal{T}. \qquad (6)$$

*Procedure S3:. Model of storage technologies*

The storage technologies $s \in \mathcal{S}$ are modeled by a power-rated capacity $C^{\mathrm{P}}_{s,n}$ [GW] and an energy-rated capacity $C^{\mathrm{E}}_{s,n}$ [GWh]. We do not apply a power-energy ratio (as, e.g.,[57]), but the balance between $C^{\mathrm{P}}_{i,n}$ and $C^{\mathrm{E}}_{i,n}$ and the charge and discharge efficiency $\eta^{\mathrm{c}}_s = \eta^{\mathrm{d}}_s = \eta_s$ determine the temporal behavior of the storages. The storage energy balance is time-coupled with the storage level $E_{s,n,t}$ and the self-discharge coefficient $\delta_s$ with $\Delta t = 1$h for $s\in\mathcal{S}, \forall n\in\mathcal{N}$:

$$E_{s,n,t} = (1-\delta_s\Delta t)E_{s,n,t-1} + F^{\mathrm{c}}_{s,c,n,t}\eta_s - F^{\mathrm{d}}_{s,c,n,t}/\eta_s \qquad \forall t\in\mathcal{T}\setminus\{t_0\}, \qquad (7)$$

with

$$E_{s,n,t} \leq C^{\mathrm{E}}_{s,n} \qquad \forall t\in\mathcal{T}, \qquad (8)$$

$$F^{\mathrm{c}}_{s,c,n,t} \leq C^{\mathrm{P}}_{s,n} \qquad \forall t\in\mathcal{T}, \qquad (9)$$

$$F^{\mathrm{d}}_{s,c,n,t} \leq C^{\mathrm{P}}_{s,n} \qquad \forall t\in\mathcal{T}. \qquad (10)$$

In this study, we assume no self-discharge for the two storage technologies: pumped hydro storage and natural gas storage ($\delta_s = 0$[58]). The initial storage level $E_{s,n,t_0}$ can be set to a share $E^0_{s,n}$ of $C^{\mathrm{E}}_{s,n}$:

$$E_{s,n,t_0} = E^0_{s,n} C^{\mathrm{E}}_{s,n}. \qquad (11)$$

The annual periodicity of storages can be enforced. In that case, Eq. (7) is formulated for $t_0$ and the last time step $t^{\mathrm{end}}$:

$$E_{s,n,t_0} = (1 - \delta_s \Delta t) E_{s,n,t^{\mathrm{end}}} + F^{\mathrm{c}}_{s,c,n,t_0} \eta_s - F^{\mathrm{d}}_{s,c,n,t_0} / \eta_s. \tag{12}$$

If disabled, the storage periodicity constraint is skipped.

*Procedure S4:. Time series aggregation*

To reduce the computational complexity, we aggregate the hourly resolved input data to 1400 representative time steps, using the time series aggregation package tsam 2.1.0[35]. The time-dependent input data are the capacity factors of conversion technologies and the electricity and heat demand. The aggregation to 1400 representative time steps allows for a detailed depiction of the temporal behavior of the energy system. As proof of concept, we optimize the central scenario of no Russian gas available under collaborative behavior with a fully resolved time series (8760 time steps). The total system cost is increased by 0.62 % in comparison to the aggregated case. The total reduced demand for all countries and final energy carriers is increased by 1.5 %. The k-means aggregation algorithm smoothens extreme values[35]. However, in this study, a full time representation increases the maximum demand reduction in any time step by only 2.7 %. Similar deviations can be expected for the other scenarios. The scenario of selfish behavior cannot be optimized with a fully resolved time series because of the computational intractability.

The temporal representation of storage technologies is challenging because the storage constraints are time-coupled, thus the sequence of time steps must be preserved[31]. To enable both the modeling of short- and medium-term storage, e.g., pumped hydro storage, and long-term storage, e.g., natural gas storage, we present a novel formulation, where the energy-rated storage variables are resolved on a different time sequence, broadly based on[31]. In particular, each change in the aggregated time sequence for power-rated variables yields an additional time step for the energy-rated storage variables. Assume the aggregation of the exemplary full time index $\mathcal{T} = [0, ..., 9]$ to four representative time steps $\mathcal{T}_\sigma = [0, ..., 3]$ with the sequence $\sigma$ for power-rated variables:

$$\sigma = [0, 0, 1, 2, 1, 1, 3, 3, 2, 0]. \tag{13}$$

The resulting sequence for energy-rated storage variables $\sigma^{\mathrm{s}}$ of the storage time steps $\mathcal{T}_{\sigma^{\mathrm{s}}} = [0, ..., 6]$ is then:

$$\sigma^{\mathrm{s}} = [0, 0, 1, 2, 3, 3, 4, 4, 5, 6]. \tag{14}$$

While this formulation enables both the short-term and long-term operation of storages, it increases the number of time steps $|\mathcal{T}_{\sigma^{\mathrm{s}}}|$ and thus the number of variables. Since the time series aggregation does not always yield the same sequence of time steps of power-rated variables, the number of time steps for energy-rated time steps varies between scenarios. The aggregation to $|\mathcal{T}_\sigma| = 1400$ representative time steps of the power-rated variables leads to between $|\mathcal{T}_{\sigma^{\mathrm{s}}}| = 2287$ (scenario with selfish behavior) and $|\mathcal{T}_{\sigma^{\mathrm{s}}}| = 2347$ (scenario with mild winter with high energy savings) time steps for energy-rated variables.

*Procedure S5:. Scope of case study*

We model the European energy system as a network representation, consisting of 28 nodes (countries) and edges between them (aggregated power lines and natural gas pipelines).

1. Austria (AT)
2. Belgium (BE)
3. Bulgaria (BG)
4. Switzerland (CH)
5. Czech Republic (CZ)
6. Germany (DE)
7. Denmark (DK)
8. Estonia (EE)
9. Greece (EL)
10. Spain (ES)
11. Finland (FI)
12. France (FR)

13. Croatia (HR)
14. Hungary (HU)
15. Ireland (IE)
16. Italy (IT)
17. Lithuania (LT)
18. Luxembourg (LU)
19. Latvia (LV)
20. The Netherlands (NL)
21. Norway (NO)
22. Poland (PL)
23. Portugal (PT)
24. Romania (RO)
25. Sweden (SE)
26. Slovenia (SI)
27. Slovakia (SK)
28. United Kingdom (UK)

We choose a spatial resolution at national level which for most countries corresponds to the control and bidding zones for transmission system operators in the electricity and gas sector. A finer spatial resolution is avoided due to computational limitations. We decide the trade-off between temporal and spatial resolution in favor of the temporal resolution because of the importance to capture the most critical hours in which the electricity and heat supply cannot be guaranteed. We acknowledge that a higher spatial resolution improves the representation of renewable generators. ENTSOG identified that cross-border gas flow capacities between countries are more prone to congestion in case of a Russian gas disruption than the internal interconnections of individual countries[19]. Thus for this study, a national resolution is sufficient to resolve the European gas flow network on a national level.

Tables S1 and S2 summarize the conversion, transport, and storage technologies included in this study. We assume linear time- and location-independent conversion efficiencies. The capacities are rated by the output carrier, i.e., capacities of electricity generation technologies are rated by their electric power, and capacities of heat generation technologies by their thermal power. In this study, we prohibit additional investments, thus the existing capacities cannot be expanded.

Note that we do not model the electricity and gas network in detail but use a flow network representation with aggregated cross-border capacities. The connections of transport technologies are assumed to span between the centroids of the connected country nodes. Thus, the distances are in general longer than the real cross-border power lines and gas pipelines. The validation of this modeling approach is out of the scope of this paper.

*Procedure S6:. Infrastructure - gas sector*

There are three technologies that are exclusively tied to natural gas:

1. Liquefied natural gas (LNG) terminals (conversion technology)
2. Natural gas pipelines (transport technology)
3. Natural gas storage (storage technology)

We assume that LNG terminals regasify LNG to natural gas with an efficiency of 100% and that the supply of LNG is a continuous process. The data for existing capacities of LNG terminals is obtained from the SciGrid GIE dataset[8]. Other SciGrid datasets such as IGGIELGN showed inaccuracies in the reported data[9]. In the scenarios without Russian gas, we include those LNG import capacities that have been recently built or will start operation beginning of 2023 (Table S3). We set the capacity factor of those capacities that will start their operation in 2023 to zero before January 1, 2023, so that they cannot be used before. We acknowledge that some of these capacities would have been built even without the Russian gas disruption, however, in particular the fast completion of construction is due to the energy crisis[4]. Thus, it is appropriate to assume that they would not be available in 2023 in a scenario with Russian gas available.

The aggregated cross-border capacities of natural gas pipelines between the countries in the case study are obtained from the ENTSOG/GIE System Development Map 2021/2022[17]. The cross-border capacities are directional and can thus show different capacities depending on the flow direction. The technical capacities reported by ENTSOG are defined as the maximum firm capacity that the transmission system operator can offer to the users, which already accounts for system integrity and operational requirements of the network[15]. Hence from an operational perspective, the cross-border flow capacities are able to support the cross-border flows obtained in this study. Because of data inaccuracies, the SciGrid datasets are not used. Newly commissioned pipeline capacities that are not included in the System Development Map 2021/2022[17] are extracted manually from the ENTSOG Transparency Map[20] and listed in Table S4. Note that the natural gas pipeline between EL and IT passes through Albania, and is thus not listed in[17] but added manually from[20].

The existing capacities of natural gas storages (power-rated and energy-rated) are obtained from the SciGrid IGGIELGN dataset[9]. Significant data inaccuracies are observed for data points that are only found in the INET dataset, especially for the UK. Thus, we exclude those storages with only an INET source id. The initial storage level for the first time step of the optimization period is obtained from AGSI for November 1, 2022[1].

*Procedure S7:. Energy carrier availability - gas sector*

In each country, there are three potential gas sources (see Fig. S2):

1. Pipeline imports
2. LNG imports
3. Domestic production

We do not model the pipelines connecting the 28 investigated countries and the outside world as cross-border capacities but limit the import availability of gas by the pipeline capacity connecting each country. The hourly import capacities are calculated from the SciGrid IGGIELGN dataset[9]. We manually add two additional import capacities from Turkey to BG (24 GW) and EL (14.583 GW) from the ENTSOG Transparency Map[20] since they are not contained in the IGGIELGN dataset.

The gas availability does not only depend on the pipeline import capacities but also the production capacities of the exporting regions. Table S5 lists the gas availability as projected by ENTSOG's and ENTSO-E's Ten Year Network Development Plan (TYNDP) 2022[16]. We choose the base availability to be the min, mean, or max of the provided range to resemble historic values[30]. The additional availability because of increased imports to compensate for the missing Russian gas imports is obtained from[30]. With Russian gas available, the gas import availabilities are not increased. The gas availability is allocated to the connected countries by their pipeline import capacity[9,20] and the import capacities are then scaled accordingly. Note that we assume all Russian flows through Turkey to be supplied to BG (Turkstream pipeline), and all flows from Turkey, Azerbaijan, and Turkmenistan (though, 0) through Turkey to be supplied to EL (TANAP pipeline).

The domestic gas production of countries is obtained for 2020 from the Eurostat complete energy balances for gas[28]. See Table S10 for a list of all used Eurostat energy balance and energy carrier codes. NO is excluded to use the values from Table S5. The historic domestic production for CH and UK in 2020 is used[6,53]. CH does not have any domestic gas production[53]. It is reported that UK production was unusually low in 2021 and increased again in 2022 in the wake of the energy crisis[6], thus we use the 2020 values.

Without Russian gas available, we subtract the import availability of Russian gas from the availability of the connected countries (Table S5). For LNG imports, we proceed analogously and extract the availability from[16] and[30]. Since LNG imports show strong seasonal behavior[18], we constrain annual imports and not hourly imports as is done for pipeline gas. Global export capacities are expected to allow around 55 bcm (537,319 GWh) in additional LNG imports to Europe[30,36]. The import availability is allocated to the nodes

by the LNG terminal capacity and listed in Table S6. This approach neglects the strategic distribution of LNG import flows to wherever the LNG can be currently accepted. Again, without Russian gas available, we subtract the import availability of Russian LNG from the availability of the connected countries (Table S6).

*Procedure S8:. Industrial gas demand profiles - gas sector*

The industrial gas demand is calculated to include all shares of natural gas that are not consumed in the electricity or heating sector. To this end, we subtract the gas consumption in the energy transformation, commercial, and household sectors from the total supply, as reported by Eurostat for 2020[28] (Table S10). Because the UK left the EU, no data is available for the UK in 2020, thus we extract the data for 2018. We do so for all the following instances where we extract data from Eurostat. The industrial gas demand for CH is obtained from[53]. We assume a time-independent, hourly industrial gas demand, thus we divide the annual values by 8760h. The existing capacities of the industrial gas consumer technology equal the industrial gas demand in each location.

*Procedure S9:. Demand profiles - electricity sector*

We extract the measured electricity demand for each country from the ENTSO-E Transparency Platform[14] for every hour in 2021. We model the sector coupling between the electricity and heating sector by including heat pumps and electric boilers that consume electricity to produce heat (Table S1). To avoid double counting the electricity consumption for electrified heat, we calculate how much heat demand can be provided by each electrified heating technology in every country and divide it by the technologies' conversion efficiency from Table S1. We then subtract the hourly profile of electricity consumption in electrified heating from the total electricity demand.

*Procedure S10:. Existing capacity - electricity sector*

We extract the existing capacity for each country and electricity generation technology (Table S1) from the ENTSO-E Transparency Platform[14] for 2022. We observe that the reported existing capacity for CH and the Nordic countries (NO, SE, FI) is too low in[14]. Thus, we complement the existing capacities of those four countries with the Open Power System Data[42,43]. Because of better data quality, we obtain the existing capacities for run-of-river hydropower and reservoir hydropower from the JRC Hydro-power database[26].

The existing capacity of the aggregated power lines is extracted as the net transfer capacity (NTC) from the ENTSO-E Transparency Platform[14] for 2022. For those edges between countries for which no NTC is reported, we set the existing capacity to the maximum crossborder flow in 2022 from[14].

The existing capacity of pumped hydro storage is obtained from the JRC Hydro-power database[26]. It is observed that the energy-rated capacity is often too high, especially for generator units that are used both as pumped hydro storages and as reservoir hydropower plants. Hence, we limit the energy-rated capacity by the maximum dischargeable energy. To this end, we multiply the power-rated discharge capacity with the maximum discharge time of pumped hydro storages (16h[59]), divided by the discharge efficiency[50].

*Procedure S11:. Capacity factors - electricity sector*

The hourly capacity factors for solar photovoltaic, and onshore and offshore wind are obtained from Renewables.ninja, resolved on NUTS0 level[46,51]; the hourly capacity factors for run-of-river hydropower and reservoir hydropower are obtained from[56]. We observe that the capacity factors of reservoir hydropower for NO are not sufficient in January and February to ensure the supply of electricity even with Russian gas available. Thus, we overwrite the capacity factor of NO with the profile of SE. In sum, the profiles are almost equal, hence the annual quantities of dispatched electricity from reservoir hydropower are similar for both profiles.

There is ample thermal power capacity in Europe that is currently idle. However, a large share of that capacity has not generated any electricity in the past year, and thus cannot be brought back to operation in a reasonable time. From ENTSO-E Transparency Platform[14], we extract the percentage of generation

units for each conventional technology (i.e., technologies that consume an input carrier, Table S1) that dispatched at least once throughout an entire year for each country. To mitigate the impact of outliers (e.g., many of Romania's thermal power plants did not dispatch in 2021), we take the average of 2020 and 2021. Note that this is not the annual utilization factor of the aggregated capacity, but the on-off decision for each individual generation unit. We apply the percentage of dispatching units as the capacity factor of the technology, hence we assume that not the entire capacity can be utilized. In the energy crisis, several countries decided to bring back some thermal power plants and place them in reserve[3]. Thus with no Russian gas available, we assume that 25% (broadly based on[3]) of the capacity that did not dispatch in the past years can be restarted, which leads to higher capacity factors. Intuitively, a higher share of restarted power plants allows for a stronger shift of the electricity generation mix, which in turn leads to lower reductions of final energy demands. The decision to bring back idle thermal power plants is highly political and varies across countries. In particular, the decision is influenced by environmental concerns because of increased carbon emissions[48].

French electricity generation from nuclear power plants is at a 30-year low with over half of the 56 French nuclear power plants currently out of operation due to a wave of repairs and maintenance. Currently, 57% of the capacity is unavailable and it is expected that until March 31, 2023, it will be restored to around 71%[12]. We linearly interpolate between the current availability (43% on November 1, 2022) and the final availability (71% on March 31, 2023).

For all technologies in all countries not mentioned above, we assume the time-independent technical availability from[39].

*Procedure S12:. Demand profiles - heating sector*

In this study, the heating sector encompasses residential and commercial heat consumption. Industrial process heat is excluded from the heating sector and contained in the industrial gas demand. We assume a single hourly heat demand time series for each country. The annual heat demand for buildings is extracted from the EU Buildings Database[24] for space and water heating in residential and non-residential buildings for all countries except NO and CH in 2015. The hourly heat profiles for the four categories (space/water and residential/commercial heating) are obtained from the When2Heat project[49], where we assume that all residential heat demand follows the profile of multi-family houses. The heat profiles are reported in MW/TWh, hence, we multiply the heat profiles with the annual demand for each of the four categories and sum the hourly demands of the four categories to obtain the total hourly heat demand. The data on the annual heat demand for each of the four categories is missing for NO and CH, thus, we assume that the ratio of heat demand for the four categories is equal to that of SE and AT, respectively. We scale the heat demand of each category to the total heat demand in 2020; for NO the total heat demand is obtained from[10] and for CH from[52]. The heat profiles for the four categories are reported in[49] for NO and CH. We neglect cooling demands in this study.

*Procedure S13:. Existing capacity - heating sector*

It is particularly challenging to retrieve data on the existing capacities of heating technologies. The Heat Roadmap Europe (HRE) project[45] provides values for the existing heating capacities, however, this information is limited to 14 European countries and the data is only available for 2015 and a broad aggregation of technology types. Hence, we model the heating capacities based on the generated heat in 2020, as reported in the complete energy balance by Eurostat[28]. We assume that the capacity share $r^{\mathrm{tot}}_{i,n}$ of a heating technology $i \in \mathcal{I}$ in country $n \in \mathcal{N}$ is proportional to its share in historic heat generation. Following[45], we assume a 10% excess capacity, i.e., in sum, the total capacity of all technologies in a country amounts to 110% of the country's peak heat demand.

In calculating the technology share, we differentiate between heat generated onsite and heat supplied from offsite district heating. We acknowledge that heat from district heating technologies is often generated in combined heat and power (CHP) plants, however, in this study, we model the technologies separately. Hence as an example, district heat generated from burning coal might come from a hard coal CHP plant

but this technology is modeled as a hard coal electric power plant and as a hard coal boiler in this study (Table S1). Furthermore, we aggregate the onsite and offsite capacities, e.g., an onsite hard coal boiler and an offsite district heating hard coal boiler are aggregated to hard coal boilers.

We extract the heat generated onsite from the final consumption energy balance in other sectors (residential and commercial) and the heat generated offsite from the gross heat production energy balance[28]. We allocate peat, peat products, and nuclear heat to hard coal boilers, and manufactured gases to gas boilers. While the allocated fuels are in general similar, nuclear heat is obviously strongly different from heat from hard coal. However, the heat generation technology of offsite hard coal and nuclear power plants is similar, and nuclear heat only accounts for 0.17% of heat generation (exclusively offsite). Biomass boilers entail both solid and gaseous biomass. The final consumption energy balance is given in quantities of the input carriers, while the gross heat production is given in quantities of generated heat. Thus, we multiply the final consumption energy balance with the conversion efficiencies from Table S1 to obtain the generated heat in the residential and commercial sectors. We scale the gross heat production energy balance to match the derived heat consumed in the residential and commercial sectors.

Electricity is the only energy carrier that is widely consumed for other services than heating and cooling in the residential and commercial sectors. Thus, we subtract that share of residential electricity consumption that is not used for heating and cooling, obtained from Eurostat's disaggregated final energy consumption in households energy balance (NRG_D_HHQ)[29]. There are two heat generation technologies that consume electricity, namely, heat pumps and electric boilers. The ambient heat (heat pumps) energy carrier in the complete energy balances[28] does not yield a realistic share of heat pumps in the electrified heat generation. Thus, we utilize the IDEES 2015 database[38] to obtain the ratio between electric boilers (conventional electric heating) and heat pumps (advanced electric heating) for 2015. We assume that NO has the same ratio between electric boilers and heat pumps as SE. Furthermore, we assume that CH has the same technology share for all technologies as AT.

The existing capacities of the heat generation technologies for all 28 countries are listed in Table S7. In general, the presented approach yields total capacities which are in line with those available in the HRE project[45], in comparison to HRE: -11% for DE, +0.9% for FR, +8% for IT.

*Procedure S14:. Fuel substitution - heating sector*

In contrast to electricity generation, heat generation cannot be easily substituted by another fuel even if there is idle capacity available. Indeed, most heat is generated onsite and usually buildings generate heat with only one energy carrier (monovalent operation). Furthermore, the heat cannot be shared with other buildings but must be consumed on-site where it is produced. However, in this nationally aggregated case study, we cannot distinguish between different buildings. Thus, we apply a capacity factor to all heat generation technologies so that the usable capacity of the heat generation technologies is seemingly reduced in times of lower heat demand. As an example, on an autumn day with a heat demand of half the peak demand, we reduce the usable capacity of the onsite heat generation technologies by 50%. Hence, even though not the entire nominal capacity is utilized at that moment, the capacity cannot be used to substitute the heat generation in another building but can only supply the heat demand of the building in which it is installed. We neglect that some buildings are supplied by two onsite heat technology sources, such as a gas boiler and a heat pump. By applying a capacity factor to the heating technologies, we can implicitly prevent heat substitution between individual buildings without modeling each building individually.

In district heating networks, the use of multiple fuels (bivalent operation) is more common than in onsite heat generation. However, especially small district heating networks are often operated by a single fuel source, and even in bivalent district heating networks fuel supply constraints make it difficult to substitute other technologies. Based on estimates by experts, we assume that $r^{\mathrm{sub}} = 25\%$ of district heating capacities can always operate at nominal capacity to substitute heat generation in the district heating network.

For the heating technology $i$ in each country $n \in \mathcal{N}$, the total technology share $r_{i,n}^{\mathrm{tot}}$ is the sum of the share

of the capacity producing heat on-site $r^{\mathrm{on}}_{i,n}$ and supplying heat via the district heating network $r^{\mathrm{DH}}_{i,n}$:

$$r^{\mathrm{tot}}_{i,n} = r^{\mathrm{on}}_{i,n} + r^{\mathrm{DH}}_{i,n} \qquad \forall n \in \mathcal{N}. \tag{15}$$

The total share of all heating capacities that supply heat via district heating is $r^{\mathrm{DH}}_{n}$, which is the sum of the capacity share of all technologies producing heat off-site $r^{\mathrm{DH}}_{i,n}$:

$$r^{\mathrm{DH}}_{n} = \sum_{i\in\mathcal{I}} r^{\mathrm{DH}}_{i,n} \qquad \forall n \in \mathcal{N}. \tag{16}$$

For those technologies that cannot substitute the heat generation of other technologies, the capacity factor $CF^{\mathrm{nosub}}_{i,n,t}$ is the hourly relative heat demand (hourly heat demand $D_{t,n}$ divided by the peak heat demand $\max_{t\in\mathcal{T}}\left[D_{t,n}\right]$) for each hour $t \in \mathcal{T}$ with $|\mathcal{T}| = 8760$ h:

$$CF^{\mathrm{nosub}}_{i,n,t} = \frac{D_{t,n}}{\max_{t\in\mathcal{T}}\left(D_{t,n}\right)} \qquad \forall t \in \mathcal{T}, \forall n \in \mathcal{N}. \tag{17}$$

The capacity factor for the onsite capacity $CF^{\mathrm{on}}_{i,n,t}$ is:

$$CF^{\mathrm{on}}_{i,n,t} = r^{\mathrm{on}}_{i,n} CF^{\mathrm{nosub}}_{i,n,t} \qquad \forall t \in \mathcal{T}, \forall n \in \mathcal{N}, \tag{18}$$

since we assume that no heat produced on-site can be substituted by other technologies, as discussed above.

The capacity factor for the capacity supplying the district heating network is composed of the share that can substitute $r^{\mathrm{sub}}$ and the share that cannot substitute $(1 - r^{\mathrm{sub}})CF^{\mathrm{nosub}}_{i,n,t}$:

$$CF^{\mathrm{DH}}_{i,n,t} = \min\left(r^{\mathrm{DH}}_{i,n}\left[r^{\mathrm{sub}} + (1 - r^{\mathrm{sub}})CF^{\mathrm{nosub}}_{i,n,t}\right], r^{\mathrm{DH}}_{n} CF^{\mathrm{nosub}}_{i,n,t}\right) \qquad \forall t \in \mathcal{T}, \forall n \in \mathcal{N}. \tag{19}$$

By limiting the capacity factor to the upper bound $r^{\mathrm{DH}}_{n} CF^{\mathrm{nosub}}_{i,n,t}$, we ensure that technology $i$ can at most substitute the entire heat supplied by the district heating network. Hence, buildings with access to the district heating network cannot substitute heat in buildings with onsite heat generation units. The combined capacity factor $CF_{i,n,t}$ is the sum of $CF^{\mathrm{on}}_{i,n,t}$ and $CF^{\mathrm{DH}}_{i,n,t}$, normalized to 1 by $r^{\mathrm{tot}}_{i,n}$:

$$CF_{i,n,t} = \frac{CF^{\mathrm{on}}_{i,n,t} + CF^{\mathrm{DH}}_{i,n,t}}{r^{\mathrm{tot}}_{i,n}} \qquad \forall t \in \mathcal{T}, \forall n \in \mathcal{N}. \tag{20}$$

In effect, the higher $r^{\mathrm{DH}}_{i,n}$, the more heat generation can be substituted by technology $i$.

*Procedure S15:. Energy carrier availability*

In this study, we investigate the potential shift of electricity and heat generation to additional technologies. The amount of electricity and heat that can be generated from the various input energy carriers is constrained (i) by the existing technology capacity and (ii) by the availability of the energy carriers in each country. The availability of pipeline gas and LNG is described in Procedure S7. In this model, none of the other input carriers in Table S1 except electricity can be transported between countries but only imported directly at the node. We acknowledge that there exists a transport system for most other energy carriers in Europe.

To predict realistic quantities of shifted electricity and heat generation, we constrain the annual import availability for the energy carriers hard coal and lignite, biomass, oil, and waste. Because of the low operational costs for nuclear power plants (Table S9), nuclear power plants are already dispatching before gas turbines and other thermal power plants. Hence, the gas shortage does not lead to a significant increase in electricity generated from nuclear (see Fig. 4). Thus, we refrain from limiting the import availability of nuclear to not over-constrain the optimization problem.

To allow the optimization model to decide when to import how much, we constrain the annual import availability but leave the allocation to each hour to the optimizer. For all constrained energy carriers, we extract the transformation input in the energy sector and the final consumption in the commercial and residential sectors from Eurostat for 2020[28]; the sum of the transformation input and the final consumption is the annual availability. We assume that CH has the same annual availability as AT. For all energy carriers except hard coal and lignite, we assume that the availability does not change due to the energy crisis. We acknowledge that the imports of Russian oil make up a significant share of European oil consumption, however, in this study, we focus primarily on the gas sector and oil does not play the main role in European electricity and heat generation.

It is reported that coal imports from outside Europe increased by 35% during the energy crisis[2]. It is not specified if this refers to hard coal or lignite, thus we apply the increase to both energy carriers, albeit most lignite is produced domestically.[37] assume that domestic lignite production can increase by 15%, whereas domestic hard coal production stays flat. To calculate the increase in hard coal and lignite availability, we obtain the primary production and imports of hard coal and lignite from Eurostat for 2020[28], increase the production and import capacity by the share stated above, and scale it to the transformation input in the energy sector and the final consumption in the commercial and residential sectors. The availability of hard coal and lignite is on average increased by 20.6% and 15.1%, respectively. The national ratio between imports and primary production determines the increase for each country.

*Procedure S16:. Fuel and operational costs*

Table S8 summarizes the prices for importing the energy carriers that are consumed in electricity and heat generation technologies. This study does not attempt to model the impact of the economic feedback as a result of the energy crisis, where fossil fuel prices reached unprecedented levels[25]. Thus, we keep the fuel prices at the level before the strong increase in late 2021. The prices for uranium, hard coal and lignite are extracted from[39] for 2015. The uranium price is assumed to remain constant, whereas the hard coal and lignite price are scaled to 2021 values by the evolution from[7]. The prices for oil (heavy oil) for 2021 are extracted from[21] and for biomass are extracted from[34].

The natural gas and LNG prices are extracted for each country for the second quarter of 2021[23]. We calculate the seasonal variation of the wholesale gas price for each country from[22]. The variation is obtained for 2017 because it is the last year in which the gas price at the beginning and the end of the year are almost equal. Since the LNG, coal, and oil prices in general follow the gas price, we multiply the yearly averages with the yearly variation of the gas price.

Table S8 lists the carbon intensities of each fuel. Note that we allocate the greenhouse gas emissions of consuming a fuel in a conversion technology to the fuel and not to the technology. By doing so, we inherently incorporate that technologies with lower conversion efficiencies emit more greenhouse gases. We assume a carbon price of 57 Euro/ton (average price of EU ETS in 2021[55]). Table S9 lists the variable operation costs (OPEX) of the electricity generation technologies and the LNG terminals. Fuel costs are not contained in the variable OPEX.

*Procedure S17:. Filling gas storages in preparation for next winter*

Next year, Europe could potentially be in a worse situation than it is in right now, without a summer with Russian gas available to fill gas storages. During the summer of 2022, significant import capacities of Russian gas were available, which enabled Europe to fill its gas storages by almost 95% until November 1, 2022, in preparation for the heating season[1]. It is unlikely that Russian gas imports will return in the summer of 2023, and Europe will need to prepare for the following heating season with significantly less gas to fill the storages. To ensure that countries are well prepared for the winter of 2023, all main results analyze the case in which the gas storage levels at the end of the year match the initial storage levels for each country.

The figure in this section shows the evolution of the European gas storage level (upper plot) with Russian gas available (light blue (iv)) and without Russian gas available (dark blue (i)) in the “winter as usual”

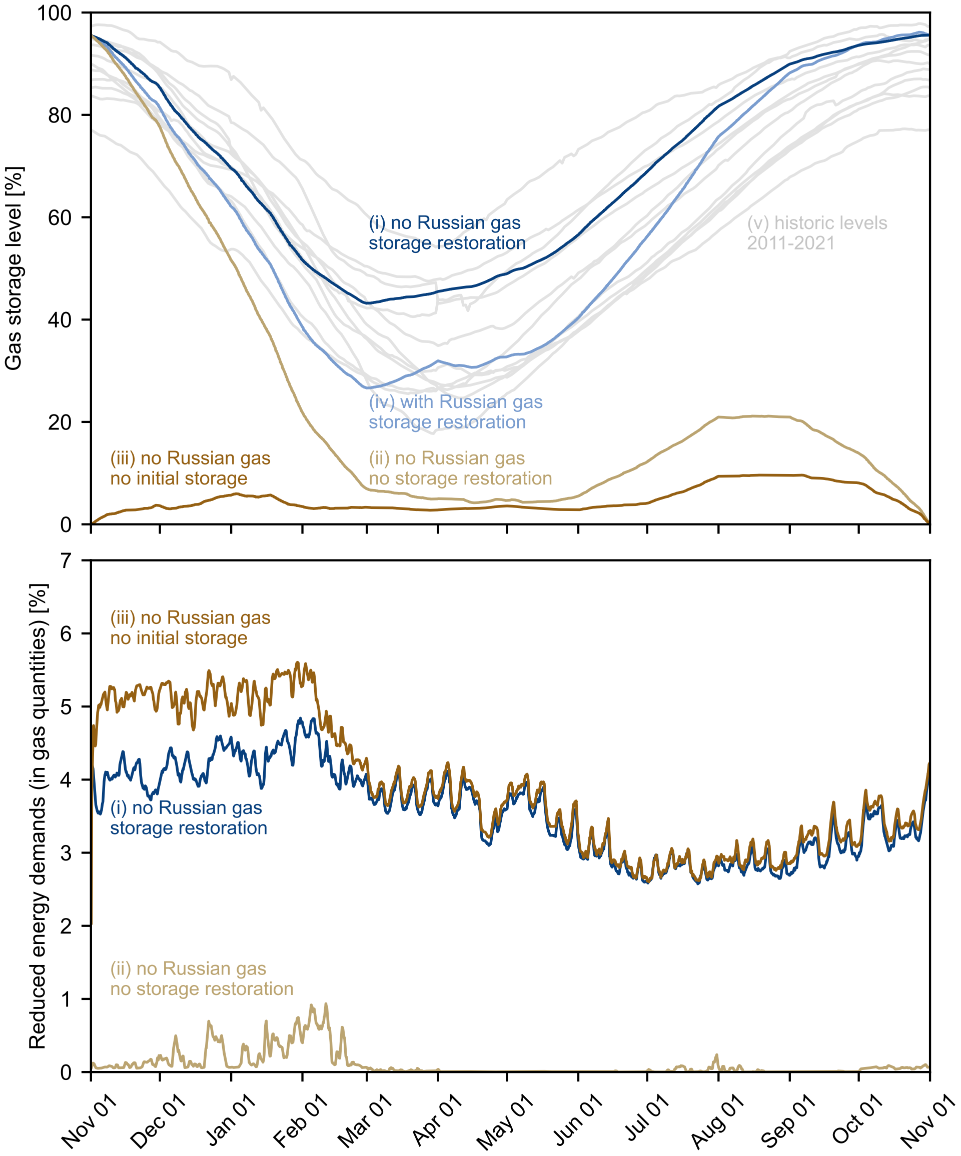


Yearly evolution of gas storage level (upper plot) and reduced energy demands (lower plot), aggregated for all countries. Comparison of: this year when preparing for next winter by restoring the initial storage levels (dark blue (i)); this year when not preparing by using all available gas in the storages and neglecting storage restoration (light brown (ii)); next year when starting with empty storage levels (dark brown (iii)). The evolution of gas storage levels (upper plot) is compared with the scenario with Russian gas available (light blue (iv)) and with historic storage levels from 2011-2021 (grey (v)[1]). Reduced energy demands (lower plot) are converted to gas quantities, divided by the demand (in gas quantities), and shown as the daily rolling mean.

scenario in comparison to historic storage levels in Europe from 2011 until 2021 (grey (v))[1]. Both scenarios fall well within the interval of historic values. Gas storages are more deeply discharged when Russian gas is available because then sufficient import quantities are available in summer to charge the storages faster and restore the initial levels.

If Europe did not plan ahead for the next winter and use the stored gas to avoid any energy demand reduction in the coming year (light brown (ii)), the gas storages would be empty at the beginning of the

following heating season (dark brown (iii)). The lower plot of the figure shows the yearly evolution of reduced energy demands (as a fraction of the total energy demand in hourly resolution, shown as the daily rolling mean) for three scenarios without Russian gas available:

(i) this year with the restoration of the initial storage level (dark blue);

(ii) this year with no restoration of the initial storage level (light brown);

(iii) next year with an empty initial storage level (dark brown)

The demand reductions are converted to gas quantities. In all scenarios, the most critical period with the highest demand reductions is the heating period from November (in this case study until the end of February). If the initial storage level is not restored, more gas can be consumed, particularly during the heating season, which results in significantly lower demand reductions. However, in the following year, Europe would then enter the heating season with empty storages. Gas availability would not be sufficient to supply the final energy demands, resulting on average in 40% higher demand reductions during the heating season with respect to the scenario in which the initial level is restored.

## Supplemental references


[1] AGSI, 2022. Gas Infrastructure Europe. URL: https://agsi.gie.eu/.

[2] Bloomberg, 2022. Europe Snaps Up Coal From Abroad to Fill Gap Left by Russia. URL: https://www.bloomberg.com/news/articles/2022-07-05/coal-imports-pour-into-energy-starved-europe-from-around-globe#xj4y7vzkg.

[3] Brown, S., 2022. Coal is not making a comeback: Europe plans limited increase. Technical Report. Ember Climate. URL: https://ember-climate.org/insights/research/coal-is-not-making-a-comeback/.

[4] Clean Energy Wire, 2022. Ukraine war puts plans for German LNG terminals back on the table. URL: https://www.cleanenergywire.org/factsheets/liquefied-gas-does-lng-have-place-germanys-energy-future.

[5] Danish Energy Agency, 2022. Technology Data for Generation of Electricity and District Heating. Technical Report. Danish Energy Agency. URL: https://ens.dk/en/our-services/projections-and-models/technology-data/technology-data-generation-electricity-and.

[6] Department for Business Energy and Industrial Strategy, 2022. Digest of UK Energy Statistics (DUKES). URL: https://www.gov.uk/government/collections/digest-of-uk-energy-statistics-dukes.

[7] Destatis, 2022. Data on energy price trends. Technical Report. Destatis. URL: https://www.destatis.de/EN/Themes/Economy/Prices/Publications/Downloads-Energy-Price-Trends/energy-price-trends-pdf-5619002.pdf?__blob=publicationFile.

[8] Diettrich, J., Pluta, A., Medjroubi, W., 2020. SciGRID_gas GIE_Raw. Technical Report. SciGrid. URL: https://zenodo.org/record/3985264, doi:10.5281/ZENODO.3985264.

[9] Diettrich, J., Pluta, A., Medjroubi, W., 2021. SciGRID_gas IGGIELGN. Technical Report. SciGrid. URL: https://zenodo.org/record/4767098, doi:10.5281/ZENODO.4767098.

[10] DNV, 2021. Energy Transition Norway 2021. Technical Report. Norsk Industri. URL: https://www.dnv.com/Publications/energy-transition-norway-2021-212201.

[11] Elengy, 2021. French regulated LNG terminals. Technical Report. Elengy. URL: https://www.elengy.com/images/publications/Elengy_regulated_LNG_terminals-tariffs2021.pdf.

[12] Ellefsen, E., 2022. French nuclear outages winter 2022/23 and power balance outlook. Technical Report. Energy Quantified. URL: https://www.energyquantified.com/blog/french-nuclear-outages-winter-2022-23-and-power-balance-outlook.

[13] Enerdata, 2022. Fortum and Gasgrid Finland will locate Finland's first FSRU at Inkoo port. URL: https://www.enerdata.net/publications/daily-energy-news/fortum-and-gasgrid-finland-will-locate-finlands-first-fsru-inkoo-port.html.

[14] ENTSO-E, 2022. ENTSO-E Transparency Platform. URL: https://transparency.entsoe.eu/.

[15] ENTSOG, 2017. Glossary of Existing Definitions. Technical Report. ENTSOG. URL: https://www.entsog.eu/sites/default/files/entsog-migration/publications/Tariffs/2017/170421_ENTSOG_Glossaryofdefinitions.pdf.

[16] ENTSOG, 2021a. Extra EU supply potentials TYNDP 2022. URL: https://www.entsog.eu/entsog-and-entso-es-workshop-extra-eu-supply-potentials-tyndp-2022#downloads.

[17] ENTSOG, 2021b. Transmission Capacity and System Development Maps. URL: https://www.entsog.eu/maps#transmission-capacity-map-2021.

[18] ENTSOG, 2022a. Press Release - ENTSOG publishes its Yearly Supply Outlook 2022 / 2023 in response to disruption of Russian gas supply. Technical Report July 2022. ENTSOG. Brussels. URL: https://www.entsog.eu/press-releases.

[19] ENTSOG, 2022b. Summer Supply Outlook. Technical Report. ENTSOG. URL: https://www.entsog.eu/sites/default/files/2022-04/SO0035-22_Summer_Supply_Outlook_2022_BOA_Rev8.1_220427forpublication.pdf.

[20] ENTSOG, 2022c. Transparency Map. URL: https://transparency.entsog.eu/#/map.

[21] ENTSOG, ENTSO-E, 2022. TYNDP 2022 Scenario Building Guidelines. Technical Report. ENTSOG. URL: https://2022.entsos-tyndp-scenarios.eu/wp-content/uploads/2022/04/TYNDP_2022_Scenario_Building_Guidelines_Version_April_2022.pdf.

[22] European Commission, 2019. Dashboard for energy prices in the EU and main trading partners. URL: https://energy.ec.europa.eu/data-and-analysis/energy-prices-and-costs-europe/dashboard-energy-prices-eu-and-main-trading-partners_en.

[23] European Commission, 2021. Quarterly Report Energy on European Gas Markets with focus on the impact of the global LNG market on EU gas prices. Technical Report. European Commission. Brussels. URL: https://energy.ec.europa.eu/system/files/2021-10/quarterly_report_on_european_gas_markets_q2_2021_final.pdf.

[24] European Commission, 2022a. EU Buildings Database. URL: https://ec.europa.eu/energy/eu-buildings-database_en.

[25] European Commission, 2022b. Quarterly report On European gas markets. Technical Report. European Commission. URL: https://ec.europa.eu/info/sites/default/files/energy_climate_change_environment/quarterly_report_on_european_gas_markets_q1_2022.pdf.

[26] European Commission, Joint Research Center, 2019. JRC Hydro-power database. URL: https://data.europa.eu/data/datasets/52b00441-d3e0-44e0-8281-fda86a63546d.

[27] European Parliament, 2022. Review of the EU ETS: Fit for 55. URL: https://www.europarl.europa.eu/RegData/etudes/ATAG/2022/729455/EPRS_ATA(2022)729455_EN.pdf.

[28] Eurostat, 2022a. Complete energy balance nrg_bal_c. URL: https://ec.europa.eu/eurostat/databrowser/view/NRG_BAL_C/default/table?lang=en&category=nrg.nrg_quant.nrg_quanta.nrg_bal.

[29] Eurostat, 2022b. Disaggregated final energy consumption in households - quantities nrg_d_hhq. URL: https://ec.europa.eu/eurostat/databrowser/view/nrg_d_hhq/default/table?lang=en.

[30] Fulwood, M., Honoré, A., Sharples, J., Hall, M., 2022. The EU plan to reduce Russian gas

imports by two-thirds by the end of 2022: Practical realities and implications. Technical Report. The Oxford Institute for Energy Studies. URL: https://www.oxfordenergy.org/publications/the-eu-plan-to-reduce-russian-gas-imports-by-two-thirds-by-the-end-of-2022-practical-realities-and-implications/.

[31] Gabrielli, P., Gazzani, M., Martelli, E., Mazzotti, M., 2018. Optimal design of multi-energy systems with seasonal storage. Applied Energy 219, 408–424. doi:10.1016/J.APENERGY.2017.07.142.

[32] GAZ-SYSTEM, 2022. LNG Terminal Expansion Program. URL: https://www.gaz-system.pl/en/terminal-lng/lng-terminal-expansion-program.html.

[33] Grosse, R., Binder, C., Wöll, S., Geyer, R., Robbi, S., 2017. 2017 Techno-economics for larger heating and cooling technologies. Publications Office of the European Union , 1–180URL: http://data.europa.eu/89h/jrc-etri-techno-economics-larger-heating-cooling-technologies-2017.

[34] Hoefnagels, R., Resch, G., Junginger, M., Faaij, A., 2014. International and domestic uses of solid biofuels under different renewable energy support scenarios in the European Union. Applied Energy 131, 139–157. doi:10.1016/j.apenergy.2014.05.065.

[35] Hoffmann, M., Priesmann, J., Nolting, L., Praktiknjo, A., Kotzur, L., Stolten, D., 2021. Typical periods or typical time steps? A multi-model analysis to determine the optimal temporal aggregation for energy system models. Applied Energy 304, 117825. doi:10.1016/J.APENERGY.2021.117825.

[36] International Energy Agency, 2022. Gas Market Report Q4 2022 including Global Gas Security Review 2022. Technical Report. International Energy Agency. URL: https://www.iea.org/reports/gas-market-report-q4-2022.

[37] Lau, M., Ricks, W., Patankar, N., Jenkins, J., 2022. Pathways to European Independence from Russian Natural Gas. Technical Report. ZERO Lab. URL: https://zenodo.org/record/6917456, doi:10.5281/ZENODO.6917456.

[38] Mantzos, L., Matei, N.A., Mulholland, E., Rózsai, M., Tamba, M., Wiesenthal, T., 2018. JRC-IDEES 2015: Integrated Database of the European Energy Sector. Technical Report. European Commission, Joint Research Centre (JRC). URL: https://data.jrc.ec.europa.eu/dataset/jrc-10110-10001.

[39] Mantzos, L., Wiesenthal, T., Neuwahl, F., Rózsai, M., 2019. The POTEnCIA Central scenario: an EU energy outlook to 2050. JRC Science for Policy Report , 346URL: https://publications.jrc.ec.europa.eu/repository/handle/JRC118353, doi:10.2760/32835.

[40] Neumann, F., Hagenmeyer, V., Brown, T., 2022. Assessments of linear power flow and transmission loss approximations in coordinated capacity expansion problems. Applied Energy 314, 118859. URL: https://linkinghub.elsevier.com/retrieve/pii/S0306261922002938, doi:10.1016/J.APENERGY.2022.118859.

[41] Offshore Energy, 2022. Finland: Hamina LNG terminal kicks off commercial operations. URL: https://www.offshore-energy.biz/finland-hamina-lng-terminal-kicks-off-commercial-operations/.

[42] Open Power System Data, 2020a. Data Package Conventional power plants. Version 2020-10-01. URL: https://data.open-power-system-data.org/conventional_power_plants/2020-10-01, doi:https://doi.org/10.25832/conventional{_}power{_}plants/2020-10-01.

[43] Open Power System Data, 2020b. Data Package Renewable power plants. Version 2020-08-25. URL: https://data.open-power-system-data.org/renewable_power_plants/2020-08-25, doi:https://doi.org/10.25832/renewable{_}power{_}plants/2020-08-25.

[44] Osorio, S., Pietzcker, R.C., Pahle, M., Edenhofer, O., 2020. How to deal with the risks of phasing out coal in Germany. Energy Economics 87, 104730. doi:10.1016/j.eneco.2020.104730.

[45] Paardekooper, S., Lund, R.S., Mathiesen, B.V., Chang, M., Petersen, U.R., Grundahl, L., David, A., Dahlbaek, J., Kapetanakis, I.A., Lund, H., Bertelsen, N., Hansen, K., Drysdale, D.W., Persson, U., 2018. Heat Roadmap Europe 4: Quantifying the Impact of Low-Carbon Heating and Cooling Roadmaps. Technical Report. Aalborg Universitetsforlag. URL: https://vbn.aau.dk/en/publications/heat-roadmap-europe-4-quantifying-the-impact-of-low-carbon-heatin.

[46] Pfenninger, S., Staffell, I., 2016. Long-term patterns of European PV output using 30 years of validated hourly reanalysis and satellite data. Energy 114, 1251–1265. doi:10.1016/J.ENERGY.2016.08.060.

[47] Pomorac, 2022. Povećan kapacitet uplinjavanja na LNG terminalu u Omišlju. URL: https://pomorac.hr/2022/04/11/povecan-kapacitet-uplinjavanja-na-lng-terminalu-u-omislju/.

[48] Reuters, 2022. Energy crisis seen posing 'existential threat' to climate goals. URL: https://www.reuters.com/business/energy/reuters-impact-energy-crisis-seen-posing-existential-threat-climate-goals-2022-10-03/.

[49] Ruhnau, O., Hirth, L., Praktiknjo, A., 2022. Update and extension of the When2Heat dataset. Scientific Data 6. URL: https://www.econstor.eu/handle/10419/249997, doi:10.1038/S41597-019-0199-Y.

[50] Schmidt, O., Melchior, S., Hawkes, A., Staffell, I., 2019. Projecting the Future Levelized Cost of Electricity Storage Technologies. Joule 3, 81–100. doi:10.1016/J.JOULE.2018.12.008.

[51] Staffell, I., Pfenninger, S., 2016. Using bias-corrected reanalysis to simulate current and future wind power output. Energy 114, 1224–1239. doi:10.1016/J.ENERGY.2016.08.068.

[52] Swiss Federal Office of Energy, 2022a. Energy Perspectives 2050+. URL: https://www.bfe.admin.ch/bfe/en/home/policy/energy-perspectives-2050-plus.html/.

[53] Swiss Federal Office of Energy, 2022b. Overall energy statistics 2021. URL: https://www.bfe.admin.ch/bfe/en/home/supply/statistics-and-geodata/energy-statistics/overall-energy-statistics.html.

[54] Tavares, F.B., Mitro, T., Maennling, N., Toledano, P., 2018. Manual for the Open LNG Regasification Model. Technical Report. Columbia Center on Sustainable Investment. URL: https://ccsi.columbia.edu/sites/default/files/content/docs/ourfocus/extractiveindustries/LNG-Import-Model-Manual-CCSI-2018.pdf.

[55] Trading Economics, 2022. EU Carbon Permits. URL: https://tradingeconomics.com/commodity/carbon.

[56] Trondle, T., 2020. Supply-side options to reduce land requirements of fully renewable electricity in Europe. PLOS ONE 15, e0236958. URL: https://journals.plos.org/plosone/article?id=10.1371/journal.pone.0236958, doi:10.1371/

JOURNAL.PONE.0236958.
[57] Tröndle, T., Lilliestam, J., Marelli, S., Pfenninger, S., 2020. Trade-Offs between Geographic Scale, Cost, and Infrastructure Requirements for Fully Renewable Electricity in Europe. Joule 4, 1929–1948. doi:10.1016/J.JOULE.2020.07.018.
[58] World Energy Council, 2019. Energy Storage Monitor. Technical Report. World Energy Council. URL: https://www.worldenergy.org/assets/downloads/ESM_Final_Report_05-Nov-2019.pdf.
[59] Zablocki, A., 2019. Fact Sheet Energy Storage (2019). Technical Report. EESI. URL: https://www.eesi.org/papers/view/energy-storage-2019.
